# Nanotubes from Ternary WS_2(1–*x*)_Se_2*x*_ Alloys: Stoichiometry Modulated Tunable Optical Properties

**DOI:** 10.1021/jacs.2c03187

**Published:** 2022-06-03

**Authors:** M. B. Sreedhara, Yana Miroshnikov, Kai Zheng, Lothar Houben, Simon Hettler, Raul Arenal, Iddo Pinkas, Sudarson S. Sinha, Ivano E. Castelli, Reshef Tenne

**Affiliations:** † Department of Molecular Chemistry and Materials Science, Weizmann Institute of Science, Rehovot 7610001, Israel; ‡ Department of Energy Conversion and Storage, Technical University of Denmark, DK-2800 Kgs. Lyngby, Denmark; § Department of Chemical Research Support, Weizmann Institute of Science, Rehovot 7610001, Israel; ∥ Instituto de Nanociencia y Materiales de Aragon (INMA), CSIC-Universidad de Zaragoza, 50018 Zaragoza, Spain; ⊥ Laboratorio de Microscopias Avanzadas (LMA), Universidad de Zaragoza, 50018 Zaragoza, Spain; # ARAID Foundation, 50018 Zaragoza, Spain; ¶ Department of Chemistry, Physics and Atmospheric Sciences, Jackson State University, Jackson, Mississippi 39217, United States

## Abstract

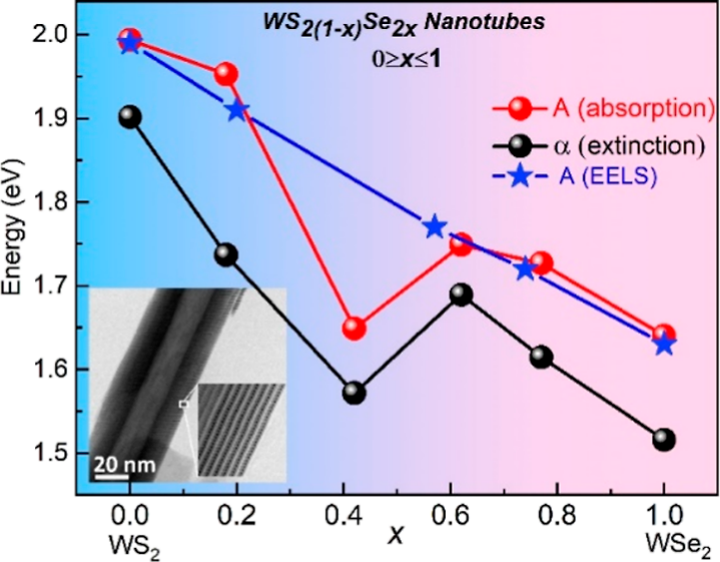

Nanotubes of transition metal dichalcogenides such as WS_2_ and MoS_2_ offer unique quasi-1D properties and numerous potential applications. Replacing sulfur by selenium would yield ternary WS_2(1–*x*)_Se_2*x*_ (0 ≤ *x* ≤ 1; WSSe) nanotubes, which are expected to reveal strong modulation in their absorption edge as a function of selenium content, *x*_Se_. Solid WO_2.72_ oxide nanowhiskers were employed as a sacrificial template to gain a high yield of the nanotubes with a rather uniform size distribution. Though sulfur and selenium belong to the same period, their chemical reactivity with oxide nanowhiskers differed appreciably. Here, the closed ampoule technique was utilized to achieve the completion of the solid–vapor reaction in short time scales instead of the conventional flow reactor method. The structure and chemical composition of the nanotubes were analyzed in detail. X-ray and electron diffractions indicated a systematic modulation of the WSSe lattice upon increasing the selenium content. Detailed chemical mapping showed that the sulfur and selenium atoms are distributed in random positions on the anion lattice site of the nanotubes. The optical excitonic features and absorption edges of the WSSe nanotubes do not vary linearly with the composition *x*_Se_, which was further confirmed by density functional theory calculations. The WSSe nanotubes were shown to exhibit strong light–matter interactions forming exciton–polariton quasiparticles, which was corroborated by finite-difference time-domain simulations. Transient absorption analysis permitted following the excited state dynamics and elucidating the mechanism of the strong coupling. Thus, nanotubes of the ternary WSSe alloys offer strong band gap tunability, which would be useful for multispectral vision devices and other optoelectronic applications.

## Introduction

Following the discovery of carbon nanotubes,^1^ nanotubes from various other layered compounds, like WS_2_,^2^ MoS_2_,^3^ BN,^4^ and so forth, were reported. The dominant mechanism for folding and seaming of the layers is the healing of the dangling bonds on the rim of the nanoparticles. It was shown that the elastic energy for folding of the layers is more than compensated by seaming and healing of the dangling bonds of the rim atoms with net energy gain. Both effective force model and ab initio calculations revealed that the overall strain energy is always positive, which means that the nanotube is stable relative to the nanoribbon of the same size but is unstable compared to an infinite layer.^5^ Furthermore, the folding energy of WS_2_ and MoS_2_ plane is about 10 times larger than that of the graphitic layers into carbon nanotubes.^6^ To comply with the large elastic energy of folding, WS_2_ (MoS_2_) nanotubes adopt larger radii than their carbon counterparts and generally come in multiwall structures;^5^ however, single-walled WS_2_ nanotubes were generated by high energy plasma radiation.^7^

**Figure 1:**
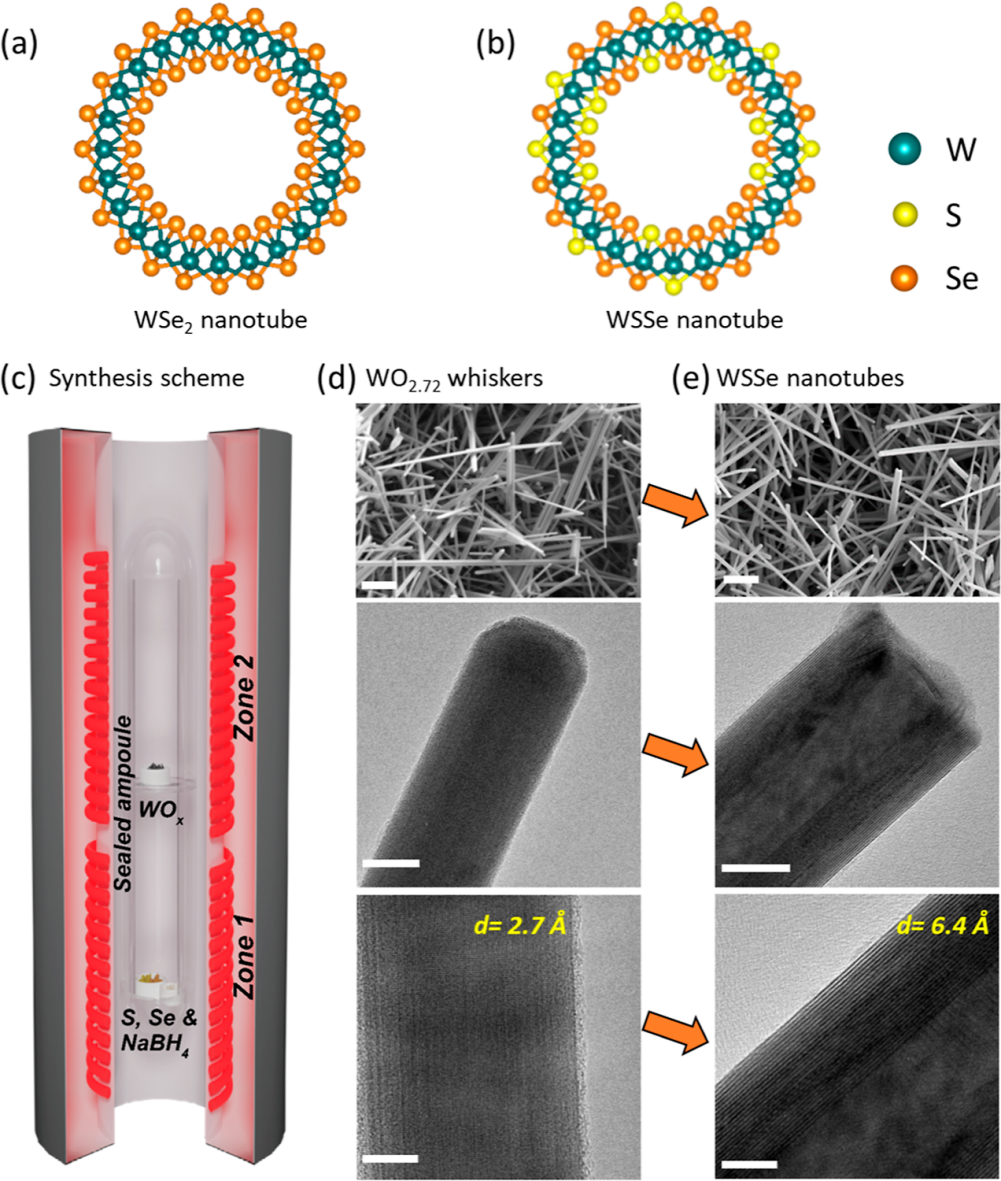
Schematic view of a single layer (a) WSe_2_ nanotube and (b) WSSe nanotube, where S and Se are distributed randomly in the lattice. (c) Synthetic scheme of the sealed ampoule method for the solid–vapor reaction in a two-zone vertical furnace. (d,e) Low and high magnification electron microscopy images revealing the conversion of WO_2.72_ nanowhiskers into WSSe nanotubes. Scale bar from top to bottom 1 μm, 20 nm, and 10 nm, respectively.

Several chemical strategies were conceived to facilitate the folding of the layers and form pure phases of nanotubes from inorganic layered compounds. One important strategy is nanotemplating. Arguably, the most successful reaction in this regard is the sulfurization of W_18_O_49_ (Mo_4_O_11_) nanowhiskers, which act as sacrificial nanotemplate for the synthesis of multiwall WS_2_,^8,9^ MoS_2_,^10^ and WSe_2_^11^ nanotubes (see Figure 1a for schematics of such nanotubes). This strategy led to the scaling-up of the production of pure WS_2_ nanotubes to semi-industrial quantities,^12^ permitting their extensive characterization and study of their physico-chemical properties. Alternatively, structure-directing agents, and in particular surface-active moieties like alkyl-amines, which form hexagonal mesophases, were found to be excellent templates for the formation of, for example, WS_2_,^13^ VO_*x*_,^14^ and nanotubes of other layered compounds. In yet another series of studies, porous alumina^15^ and carbon templates,^16^ as well as electrochemically prepared MoO_*x*_ nanotubes,^17^ were used as templates for the synthesis of MoS_2_ nanotubes. Carbon nanotubes were also used as a template for the growth of conformal MoS_2_ top layer, resulting in core–shell C@MoS_2_ nanotubes.^18^

Nanotubes from asymmetric (Janus) layered compounds, like Se–Mo–S, have been discussed in the literature quite extensively in recent years, however exclusively through ab initio calculations.^19,20^ Here, the asymmetry between the outer selenium and the inner sulfur layers elicits folding of the layers and seaming the dangling bonds at the edges, much like other asymmetric nanotubes of halloysite,^21^ immogolite,^22^ and misfit compounds.^23^ The high temperature of the reaction, though, leads to a random distribution of the sulfur and selenium in the lattice (see Figure 1b). However, in one recent case, misfit nanotubes of the kind LaS–TaSe_2_ and LaS–(TaSe_2_)_2_ with a highly asymmetric structure and large (local) dipole moment were produced, by careful control of the S/Se ratio as well as the other reaction parameters.^24^ Notwithstanding the high temperature of the reaction (825–1100 °C), the large reaction enthalpy drove it to a highly selective and specific path leading to such asymmetric nanotubes.

Mixed WS_2(1–*x*)_Se_2*x*_ nanotubes with varying composition (*x*) and random sulfur and selenium distribution in the lattice were briefly described.^25^ The nanotubes were obtained via a high-temperature sulfurization/selenization process of WO_3_ nanowhiskers in a flow reactor. Notably, the nanotubes with *x* ∼ 0.5 showed good electrocatalytic reactivity toward hydrogen production, which was attributed to improved conductivity and the high defect concentration (imperfections) in such nanotubes. In this respect, it is not unlikely that any physiochemical property will vary in a non-linear fashion with the S/Se ratio in the tubes, which warrants further study of such mixed structures.

In the present work, WS_2(1–*x*)_Se_2*x*_ (denoted as WSSe for simplicity) nanotubes with 0 ≤ *x* ≤ 1 and random sulfur and selenium distribution in the lattice are studied. The employed synthetic process is different from the one described earlier.^25^ Here, a sealed ampoule protocol was employed to obtain ternary WSSe nanotubes in a high yield with good control on the stoichiometry. Extensive structural characterization and chemical analyses of the tubes were undertaken, and their optical properties were investigated. The absorption onset of the nanotubes shrinks from 1.9 to 1.5 eV in a non-linear fashion going from WS_2_ to WSe_2_. Density functional theory (DFT) calculations of direct and quasi-direct band gap of eight-layer slabs with different compositions endorse the observed variation. Detailed spectroscopic analysis [extinction, absolute absorption, and transient absorption (TA)] revealed strong light–matter interactions, that is, strong coupling of cavity modes with excitons, resulting in polariton quasiparticles. Finite difference time domain (FDTD) simulation confirmed the strong coupling effect. TA pump–probe spectroscopy was used to elucidate the dynamics of the excited states.

## Results and Discussion

### Synthesis and Structural Investigations of WSSe Nanotubes

Nanotubes of WS_2(1–*x*)_Se_2*x*_ (0 ≤ *x* ≤ 1) were prepared via high-temperature solid–vapor reaction, using WO_2.72_ nanowhiskers as a sacrificial template. The synthesis of the oxide nanowhiskers was reported earlier and is briefly described in the Supporting Information.^26^Figure S1 reveals that they are grown as individual whiskers with the majority of them showing the size (diameter) distribution of less than 100 nm. Energy dispersive X-ray spectroscopy (EDS) chemical maps of the whiskers showed that their composition could be approximated as W_18_O_49_ (Figure S1d). The high-temperature chalcogenation reaction proceeds through a well-known Kirkendall diffusion mechanism by consuming the oxide core and subsequently forming WSSe nanotubes. Figure 1c depicts the precursor placement inside the sealed quartz ampoule and the reaction in the two-zone vertical furnace. The temperature profile during the entire process of the conversion reaction of WO_2.72_ whiskers to WSSe nanotubes was monitored and is shown in Figure S2. Several reactions were carried out to optimize the synthetic conditions for WS_2(1–*x*)_Se_2*x*_ (0 ≤ *x* ≤ 1) nanotubes. Nanotubes were obtained for the entire composition range 0 ≤ *x* ≤ 1, including pure WS_2_ and WSe_2_ ones. Positioning the WO_2.72_ whiskers separately from the S/Se source was crucial because mixing of these precursors did not yield nanotubes. Putatively, when the heated S/Se melts, it consumes the oxide template (liquid–solid reaction instead of a vapor–solid reaction), which produces WSSe flakes rather than nanotubes. Using an excess of NaBH_4_ lead to quick reduction of the oxide nanowhiskers, which resulted in WO_2_ and inhibited the chalcogenation reaction. At 840 °C, the WSSe nanotubes were found to grow seamlessly by overcoming the competition with the reduction reaction. A deviation of a few tens of degrees from 840 °C would increase the rate of reduction, which resulted in a product containing a few seamed layers of WSSe (shell) on top of the WO_2_ oxide core.

A set of scanning electron microscopy (SEM) and transmission electron microscopy (TEM) images in Figure 1d,e show WO_2.72_ nanowhiskers and their subsequent conversion into WSSe nanotubes. An extended series of SEM images of the WSSe nanotubes of different compositions (0 ≤ *x*_Se_ ≤ 1) are displayed in Figure S3. Note also that the diameter of the nanotubes is approximately the same as that of the WO_2.72_ whiskers, irrespective of their exact composition. This result is not surprising given the growth mechanism of the nanotubes, that is, chalcogenation of the WO_2.72_ nanowhiskers from outside inwards. The statistical distribution of the nanotube sizes (diameter) shown in Figure S4 reveals that the majority of the nanotubes are in the range of 80–100 nm in diameter and several microns in length. Table S1 summarizes some of the reactions carried out and the observed products with their morphologies. Although the chemistry of sulfur and selenium appears to be similar, their reactivity with respect to the WO_2.72_ solid whiskers at high temperatures varied largely and is evident in the present observations. A set of synthetic protocols used for WSe_2_ and ternary WSSe nanotubes did not yield the nanotubes in the case of pure WS_2_. Instead, very fine flakes with triangular facets were produced (Figure S5). Upon decreasing the reaction temperature (to 700 °C), the rate of reduction appears to have dominated the chalcogenation. Reducing the overall reaction time produced flakes projected outward from the whisker backbone (Figure S5c–f). These reactions indicate that sulfur is more truculent with oxide whiskers compared to selenium, and the sulfurization route deviates from the Kirkendall diffusion. Though the WS_2_ nanotube synthesis is well-known from earlier works, obtaining them in a sealed ampoule required modifying the sequence of precursors admitted into the hot zone (see Table S1). The precursors were inserted directly into the preheated furnace (without ramping the temperature) and withdrawn at once, after a short time (30 min). This sequence yielded quite a good amount of WS_2_ nanotubes (Figure S3e,f) along with WO_2_ as a byproduct (vide infra). Longer reaction times yielded WS_2_ nanotubes decorated with flakes on their surfaces (Figure S6). Reducing the thrust of sulfur during the reaction may improve the yield of the nanotubes, but it cannot be easily controlled within the sealed ampoule.

The TEM images presented in Figure 1d,e show the conversion of a WO_2.72_ nanowhisker to a WSSe nanotube. A high-resolution (HR)TEM image in the bottom panel of Figure 1d,e reveals that the atomic planes of the oxide whisker with a lattice spacing of *d* = 2.7 Å are transformed into WSSe nanotube with the interlayer spacing of *d* = 6.4 Å. Figure S7 shows a series of TEM images and selected-area electron diffraction (SAED) patterns of WSSe nanotubes with different selenium contents. The nanotubes of all compositions seem to have quite a perfect crystalline structure. The main reflections in the SAED patterns are (002) and (101) corresponding well with the XRD values (see Figure S7 and Supporting Information text). Most of the nanotubes exhibit diffraction patterns with unique helicity; that is, all the nanotube walls are orientated in the same direction. In some cases of intermediate composition, multiple helicities were encountered. In these cases, several orientations in the diffraction pattern can be attributed to individual nanotube’s wall (Figure S7).

SEM–EDS and scanning TEM (STEM)–EDS were carried out to estimate the average chemical composition of the nanotubes. It was found that the elemental distribution was uniform along the entire length of the nanotubes. The compositions were expressed in terms of Se concentration in the sample, that is, *x*_Se_ for simplicity (*x*_Se_ = 2*x*, see Table S2). The actual chemical composition (*x*_Se_) of the nanotubes was found to be quite different from the concentration of the chalcogen atoms in the precursor mixture (*X*_Se_; *X*_S_, Table S2). Hence, the actual composition in terms of selenium concentration (*x*_Se_) obtained from EDS is used here to express the stoichiometry. Figure S8 shows the SEM–EDS analysis of WSe_2_ and WSSe (*x*_Se_ = 0.42) nanotubes, respectively. Table S2 summarizes the average chemical composition (*x*_Se_) obtained from EDS analysis along with the nominal compositions (*X*). Comparing the ratio between the Se to S content in the precursor to the composition of the nanotubes in the product, it is clear that sulfur is much more truculent than selenium. The difference in reactivity between sulfur and selenium can be attributed to the higher ionicity of the former. Therefore, sulfur reacts more vigorously with the ionic W–O bond of the nanowhiskers. In the case of pure WSe_2_, a stoichiometric amount of Se is sufficient and a solid–vapor reaction with whiskers smoothly proceeds to form the nanotubes. The formation of flakes was not observed under the present experimental conditions, indicating the chemical softness of Se. In analogy, similar reactions in a flow reactor protocol would require large throughput of Se to achieve Se-rich nanotubes. Hence, the sealed ampoule method reported here has some advantages as a proof of concept to prepare Se-rich nanotubes, while the flow reactor shows the best result for S-rich phases and is scalable.

**Figure 2:**
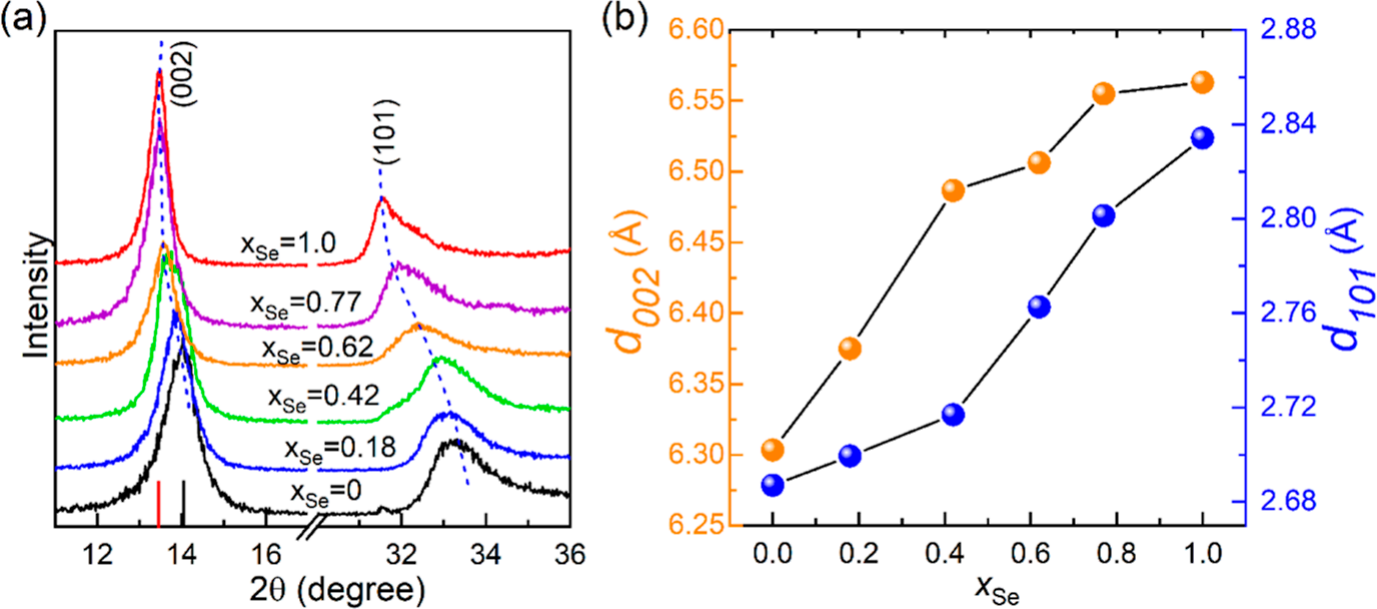
X-ray diffraction patterns of ternary WS_2(1–*x*)_Se_2*x*_ (*x* = 0 to 1) nanotubes. The position of the (002) Bragg plane of pure WS_2_ and WSe_2_ nanotubes is marked with a black and red line, respectively. The shift toward larger *d*-spacings in (002) and (101) planes upon incorporation of Se is indicated by the blue dotted lines. (b) Shift in the *d*_002_ and *d*_101_ Bragg planes as a function of Se concentration in the nanotubes.

XRD analysis was carried out for the WS_2(1–*x*)_Se_2*x*_ nanotube phases to evaluate the effect of Se content, *x*_Se_. Figure 2a displays the expanded XRD patterns of the (002) and (101) peaks of the nanotube phases with the increase in *x*_Se_, while Figure 2b summarizes the peak position as a function of *x*_Se_. The shift of the (002) peak to lower angles with the increase in *x*_Se_ represents the expansion of the interlayer spacing (*c*/2), which is attributed to the larger size of the selenium compared to the sulfur atom. Similar, but somewhat smaller, lattice expansion is observed along the *a*-axis of the unit cell. Figure S9 shows the XRD patterns (wide scan) of the different nanotube phases analyzed in this study and a product obtained relatively at lower reaction temperatures. The sharp peak (with an asterisk) is assigned to the residual monoclinic WO_2_ phase in the core of the nanotubes, which has not reacted with the chalcogen vapors. It is seen that this phase is present mostly in the pure WS_2_ and WSe_2_ nanotube phases. The competition between the reduction of WO_3_ nanoparticles and their sulfurization/selenization reaction has been discussed in some detail before.^8,27,28^ According to this study,^27^ which was carried out in a flow reactor, the temperature dependence of the two reaction rates is different, and consequently, they cross each other at around 800 °C (for sulfurization) and around 750 °C (for selenization). At these crossing points, the two reactions, that is, the rate of WO_3–*x*_ reduction by hydrogen and the chalcogenation of the reduced oxide, are synchronized and form fullerene-like nanoparticles and nanotubes. If the hydrogenation rate is too fast (<800 °C), WO_2_ forms rapidly and the sulfurization reaction is inhibited, and vice versa for temperatures higher than 800 °C. This means that the reaction conditions must be carefully optimized for each of the phases, which was also the case here. Although the temperature was the same (840 °C) throughout all the experiments, the composition of the nanotubes was controlled by varying the S and Se content in the precursors. The expanded scan of the interlayer (002) peak of WS_2_ flakes and their nanotubes is presented in the inset of Figure S9. The nanotube peak is shifted to lower angles, that is, larger interlayer spacing *d* = 6.32 Å compared to the flakes (*d* = 6.20 Å) due to the strain of the curved layers.^3^ Also, the full width at half maximum of the nanotube’s peak is substantially larger than that of the WS_2_ flakes, that is, the coherent scattering length (15–20 nm).

Raman spectroscopic analysis gave further insights into the local structure of WSSe alloyed nanotubes on a single nanotube level. The evolution of the Raman spectra of WS_2(1–*x*)_Se_2*x*_ flakes with Se content (*x*_Se_) was reported.^29,30^ If excited with a short wavelength laser (<500 nm), the peaks associated with the E_2g_ and the A_1g_ modes of WSe_2_ coalesce into a single, somewhat broadened, peak at 253 cm^–1^.^31^ For excitation wavelengths closer to the exciton resonance above 600 nm, the two peaks are separated slightly, as seen in the present case, too. The excitation wavelength used here (633 nm) is close to the A exciton of WS_2_ (630 nm) and the B-exciton of WSe_2_ (636 nm); hence, the spectra can be considered as resonance Raman. The Raman spectra of individual nanotubes with different selenium contents (*x*_Se_) were collected and are presented in Figure S10a. The strong peaks corresponding to the E_2g_ and A_1g_ modes of WS_2_ nanotubes are rather narrow and well separated (350 and 415 cm^–1^, respectively). Similarly, pure WSe_2_ nanotubes also show a strong Raman signal around 253 cm^–1^, constituting both the E_2g_ and A_1g_ modes; the splitting is nonetheless clearly visible here. In addition to characteristic Raman modes, the expanded spectrum of WS_2_ and WSe_2_ nanotubes (Figure S10b,c) shows secondary modes, which correspond to resonant Raman features. Figure S10c presents the (resonance) Raman spectrum of WS_2_ nanotubes, which was studied before.^32^Table S3 summarizes the different Raman modes of both WS_2_ and WSe_2_ nanotubes. The Raman peaks of the nanotubes with intermediate compositions were broadened but in general well resolved at any composition. The broadening of the Raman signals is due to the local structural modulations. The optical transition seems to vary nonlinearly, going from WS_2_ to WSe_2_ nanotubes. For *x*_Se_ = 0.18, the peak of the A_1g_ mode of WS_2_ is strong and relatively sharp and is shifted to 411 cm^–1^. The peak corresponding to the E_2g_ mode is broadened and becomes highly asymmetric with extended shoulder below 350 cm^–1^. At higher Se content, the original A_1g_ peak of WS_2_ at >410 cm^–1^ disappears, and the weight of the Raman spectrum is “migrated” to a highly asymmetric and broadened peak between 250 and 300 cm^–1^. The disorder in the mixed S/Se nanotubes’ lattice contributes to broadening of the peaks, but the overall picture of the Raman spectra is consistent with the literature and the other experimental data. The Raman analysis indicates that the pure WS_2_ and WSe_2_ nanotubes are structurally quite well ordered, whereas the intermediate phases show high structural modulations.

**Figure 3:**
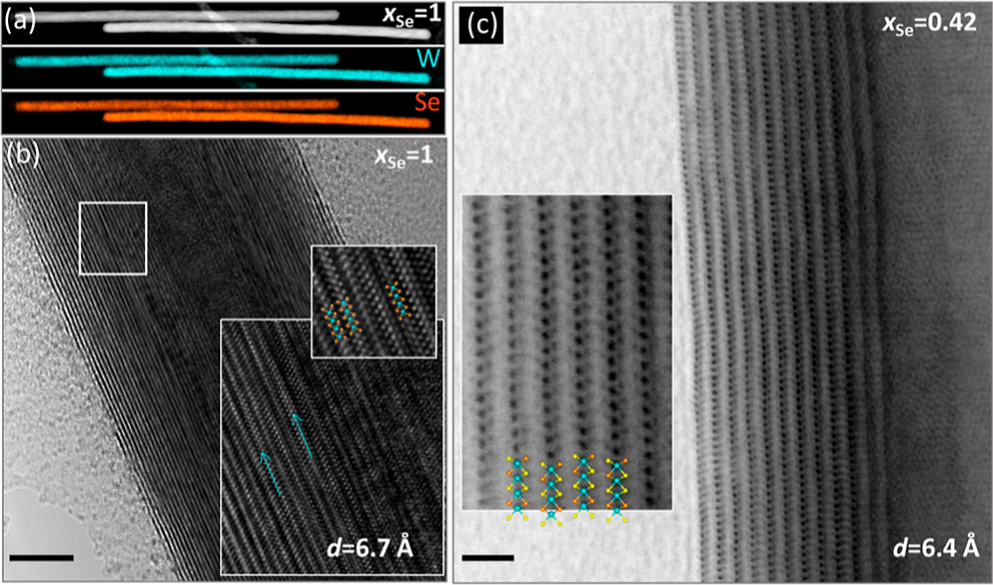
TEM analysis. (a) STEM image and STEM–EDS chemical maps of W and Se in WSe_2_ (*x*_Se_ = 1) nanotubes (diameter of the tubes is 55 nm). (b) HR-TEM image of WSe_2_ nanotube showing the stacking of layers perpendicular to the nanotube growth direction, an expanded atomic resolution image shown in the inset reveals that W and Se are in trigonal prismatic coordination. The corresponding atomic models are overlaid (W—cyan, Se—orange). (c) Atomic resolution image of WSSe (*x*_Se_ = 0.42) nanotube obtained in an aberration-corrected microscope. The chalcogen and tungsten atoms (S-yellow) in each layer are clearly seen, indicating that the alloyed nanotubes also show trigonal prismatic coordination. The atomic models of WSSe with random distribution were overlaid in the expanded image in the inset. The assignment of sulfur (yellow) and selenium (orange) atoms in this model is arbitrary, but the overall agreement with the underlying TEM image is very good.

Figure 3a shows STEM–EDS chemical mappings of two pure WSe_2_ nanotubes. The uniform selenium (orange) and tungsten (cyan) distribution in the nanotubes is clearly revealed. An HR-TEM image of one such nanotube is shown in Figure 3b; the nanotubes are perfectly crystalline, and the WSe_2_ layers are stacked perpendicular to the nanotube growth axis with an interlayer spacing of 6.6 Å. The magnified image in the inset shows W and Se atomic layers coordinated in a trigonal prismatic fashion. Figure 3c shows a bright-field HR-STEM image of a WSSe nanotube (*x*_Se_ = 0.42). The expanded view in Figure 3b,c affirms the trigonal biprism (hexagonal) coordination of the sulfur (selenium) atoms to the tungsten atom in the mixed nanotubes as well. Noticeably also, while the position of the tungsten atom of each layer is skewed with respect to its adjacent ones, the second nearest layer accommodates the same structure, typical of the 2H polytype. This direct observation, which is the first of its kind, confirms the results of the XRD analysis. One must recall, though, that because every wall of the nanotube contains a different number of atoms, the 2H structure would hold locally, only, and cannot be extended over the entire length of the nanotube. The contrast in the bright-field STEM image in Figure 3c does not differ in the inner and outer chalcogen layers of each triple layer; hence, the occupation of chalcogen sites with sulfur and selenium is random. There are cases where the signal is enhanced on the *inside* of a layer; this is expected because of the curvature, but no indication for ordering of S/Se in the nanotube, that is, Janus-type ordering,^20^ was observed (see Figure 1c). The wiggly appearance of the layers stems likely from distribution of the larger selenium atoms, which randomly form clusters and expand locally the interlayer spacing. The HR-STEM–EDS analysis of this nanotube validates a uniform distribution of the sulfur and selenium atoms across the axial and radial directions of the nanotube (Figure S11). The STEM–EDS analysis of this particular nanotube also reveals the concentrations of sulfur to be *x*_S_ = 0.6 (*x*_Se_ = 0.4), that is, much higher sulfur content than the nominal composition (*X*_S_ = 0.5), validating the SEM–EDS results.

### Optical Properties of WSSe Nanotubes

The optical properties of WSe_2_ flakes were investigated long ago.^33^ More recently, several works have been dedicated to the growth and optical and electrical properties of MS_2(1–*x*)_Se_2*x*_ (M = Mo, W)^29^ and Mo_*y*_W_(1–*y*)_X slabs, where X = S, Se.^34−36^ Because much of the density of states in the valence and conduction bands are derived from the metal d states, the band gap was found to vary (almost) linearly with the composition *x* of the chalcogen atoms.^36^ On the other hand, large deviations from linearity (bowing) with metal composition (*y*) were observed.^35^

To understand the strong light–matter interactions as a function of composition, *x*_Se_, four complementary spectroscopic techniques were employed in the present study. Low-loss electron energy loss spectroscopy (EELS) was used to investigate the absorption onset and exciton energies on individual nanotubes. Low-loss EELS is advantageous in the sense that it permits a direct correlation between the local optical signatures with the exact composition of the nanotube as determined by EDS in the TEM. The absolute absorption and extinction (absorption + scattering) measurements of a nanotube suspension bring insights into the strong coupling effects of cavity modes trapped in the nanotube with excitons, which form polaritons. The extinction spectrum is measured by simply placing the sample between the tunable light source and the detector. The recorded spectrum represents the light losses due to absorption and light scattering. The net absorption spectrum is obtained by placing the sample in the center of an integrating sphere. Finally, the TA (extinction)-pump–probe technique probes the evolution of polaritons and their excited-state dynamics in the ultrafast regime. TA is in fact a transient extinction measurement. The observed optical features were corroborated with DFT and FDTD simulations.

The band gap of WS_2_ (MoS_2_) nanotubes is smaller than that of the 2D flakes, which was attributed to the elastic energy of folding.^37^ However, owing to the relatively large diameter and small strain of the tubes, this effect is rather small (a few meV). The optical features of WS_2_ nanotubes are quite unique compared to their flakes. The nanotubes show a remarkable difference in the extinction and the net absorption spectra, which was attributed to the strong coupling effect and strong light scattering of the nanotubes.^38,39^ Owing to the large refractive index (>4), the concentric walls of the WS_2_ nanotubes with their internal cavity act as Fabry–Perot interferometers. Hence, upon illumination, optical cavity modes are confined within the nanotube, which couple with the A and B excitons (to some extent also with the C exciton). Under resonance conditions, the exciton-cavity modes lead to the formation of quasi-particles known as polaritons, which scatter light very effectively. The polaritonic features α, β, and γ observed in the extinction spectrum of WS_2_ nanotubes are red shifted with respect to the pure excitonic peaks A, B, and C in the net absorption (with integrating sphere). For instance, the lowest energy polariton α appears at 665 nm, whereas the A exciton is at 630 nm (red-shift of 100 meV).^38^ Interestingly, the dip between the α and β peaks of the extinction almost coincides with the A exciton position in the absorption spectrum. In fact, the β peak is a superposition of the high energy polariton derived from the A exciton and the low energy polariton of the B exciton hybridizing both with the same optical cavity mode.

The optical properties of WS_2_ nanotubes as a function of their diameter demonstrated that the strong coupling effect was significant in nanotubes with 80 nm diameter and above.^39^ Nanotubes with smaller diameters could not confine the light in their core and did not support cavity modes, revealing pure excitonic behavior in the extinction measurements. The strong-coupling effect was also observed in 80–100 nm thick WS_2_ flakes and discs, whereby the two faces serve as the Fabry–Perot reflectors recirculating the cavity mode.^40,41^ Strong coupling effect with higher cavity modes (*n* = 20, 21, etc.) was also found in the photoluminescence of MoS_2_ nanotubes of larger diameter (300 nm).^42^

**Figure 4:**
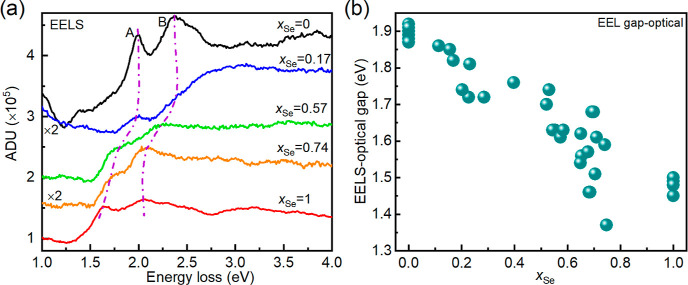
(a) Inelastic part of the low loss EEL spectrum of WSSe nanotubes with varying selenium content, *x*_Se_. The spectra were vertically translated to align them with the increasing *x*_Se_. (b) Optical gap derived from the A exciton onset of the EELS spectrum as a function of *x*_Se_ of the nanotubes. Note that more than 30 nanotubes were analyzed here.

**Figure 5:**
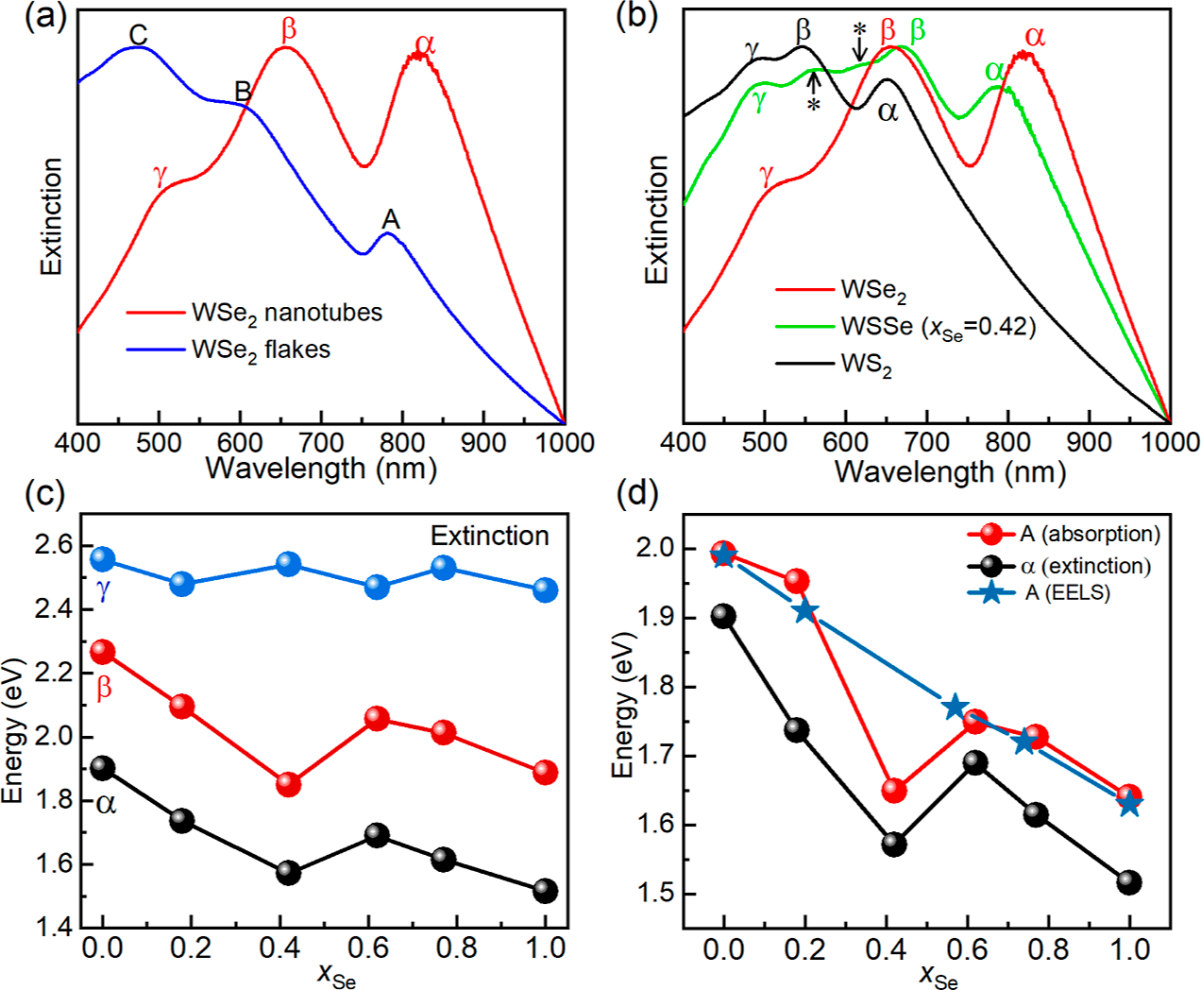
(a) Comparison between the extinction spectrum of WSe_2_ nanotubes (red curve) and WSe_2_ flakes (blue curve). Note the difference between the α, β, and γ peak positions of the tubes, and the A, B, and C (exciton) peaks of the flakes. Also, note the difference in the shape of the two curves in shorter wavelengths. (b) Extinction spectra of WS_2_, WSSe (*x*_Se_ = 0.42), and WSe_2_ nanotubes shown in black, green and red curves, respectively. All the spectral features are polaritonic in nature and exhibit redshift with respect to the net absorption spectra. (c) Variation of the α, β and γ polaritonic peak positions as a function of *x*_Se_ in the nanotubes. (d) Variation of the A exciton (from absorption measurements and EELS) and the lower polariton-α (from extinction measurements) as a function of selenium content of the nanotubes.

To measure the optical gap and exciton energies on a single nanotube, monochromated EELS has been performed in an aberration-corrected TEM. To correlate the EEL spectral feature with *x*_Se_, the composition of the nanotube subjected to EEL spectral acquisition was analyzed using STEM–EDS. Figure S12a presents an exemplary low-loss EEL spectrum of WSe_2_ nanotube, obtained as the sum of multiple spectra in a region of interest across the nanotube. Hyperspectral EEL data were recorded with a focused scanning probe with spatial and a high energy resolution (better than 90 meV). The elastic contribution to the spectrum was subtracted using the mirrored left-hand tail of the elastic zero-loss-peak. Figure S12b shows the inelastic part of the low-loss EEL signal of five different nanotubes with varying selenium content. The region of interest of the inelastic part of the low-loss spectrum of WSSe nanotubes with varying selenium content is presented in Figure 4a. The A and B excitonic features are clearly visible and are marked with purple dotted lines. Both A and B excitons shift to lower energies with increasing Se content of the nanotube. This observation is consistent with the optical properties (vide infra). The A exciton energy obtained from EELS analysis matches precisely with that of absolute absorption measurements and falls almost linearly with the increasing selenium content of the nanotubes (see Figure 5d). It is to be noted that the EELS and absolute absorbance measurements are directly related to the imaginary part of the dielectric function.^43^ The onset energy of the A exciton, that is, the optical gap, shows a significant red-shift with increasing selenium content. The low-loss EEL spectrum and the composition of more than 30 nanotubes was measured, and the onset of the A exciton peak is presented in Figure 4b as an EELS optical gap. The gap of WSSe nanotubes also falls almost linearly with increasing Se content. Interestingly, some of the WSSe nanotubes with an intermediate composition (around *x*_Se_ = 0.7) show an optical gap smaller than that of pure WSe_2_ nanotubes, which requires further investigation.

In addition, the composition dependence of the bulk plasmon energy *E*_P_ determined from EELS measurements is shown in Figure S13. The values of *E*_P_ show a decrease from 22.9 eV for WS_2_^44^ to 21.9 eV for WSe_2_^45^ nanotubes. The bulk plasmon is mainly dependent on the valence electron density,^46^ and the energy decrease, therefore, goes hand in hand with increasing lattice parameters with increasing selenium content (Figure S13b). The width of the plasmon peaks were slightly larger for nanotubes with an intermediate composition, which could stem from structural modulations in the lattice. The value of the bulk plasmon of the WS_2_ flakes (marked by a cross in Figure S13b) agrees with that of the nanotubes of the same composition.

The optical properties of suspensions of WSSe nanotubes with varying selenium content, *x*_Se_, were studied in a similar fashion as for the WS_2_ nanotubes (Figure S14).^39^ The size distribution of the WSSe nanotubes was quite narrower (80–100 nm) compared to the WS_2_ nanotubes studied before; hence, they were directly employed for optical studies with no further fractionation. Figure 5a shows the extinction spectra of WSe_2_ nanotubes in comparison with that of 2D flakes. In analogy to the spectra of WS_2_ nanotubes,^38,47^ the extinction spectrum of the WSe_2_ nanotubes is red-shifted considerably from that of the flakes. The remarkable difference between the two spectra is attributed to the strong coupling effect of optical cavity modes confined in the nanotubes (red curve) but not in the large flakes (blue curve). The absence of cavity modes and polaritonic signatures in the extinction spectrum of WSe_2_ flakes indicates a pure excitonic behavior; hence, the spectral features are referred to the pure A, B, and C excitons; that is, the extinction and the absorption spectra of the flakes are the same. The α, β, and γ polaritons of the nanotubes are red shifted compared to the A, B, and C excitons of the flakes. This situation is somewhat analogous to the scattering experiments of polarized light from WS_2_ nanodisks 55 × 130 nm in size.^40^ The scattering spectrum exhibits two new dark dip states (see Figure 3 of ref (40)) attributed to a polaritonic resonance between a dark anapole cavity mode and an exciton occurring in such nanodisk. The lower polaritonic dip (680 nm) is red shifted compared to the excitonic transition (630 nm).^40^ Moreover, while the extinction intensity of the flakes increases almost monotonously with decreasing wavelength, the extinction spectrum of the tubes shows a clear intensity decline in wavelengths shorter than the β peak, most likely due to a strong Rayleigh scattering.^38,47^ Overall, the extinction spectrum of the WSSe nanotubes for all *x*_Se_ (Figure S14a) could be interpreted in terms of a strong coupling effect between cavity modes confined in the nanotubes and the excitons.

Figure 5b shows the extinction spectra of WS_2_, WSSe (*x*_Se_ = 0.42), and WSe_2_ nanotubes. The polaritonic bands α, β, and γ are clearly visible in all the nanotubes. There is a strong red-shift of the extinction peaks going from pure WS_2_ to WSe_2_ tubes and also the extinction edge (Figure S14a). Notably, the red-shift does not vary linearly with the composition, *x*_Se_. This effect can be nicely traced by following the shift of the lower polaritons (α). While the shift in the α peak position is very large, going from WS_2_ to WSSe (*x*_Se_ = 0.42) nanotubes, it is appreciably smaller going from WSSe (*x*_Se_ = 0.42) to pure WSe_2_ nanotubes. Furthermore, the energy difference between the A and B exciton peaks is appreciably smaller for WS_2_ as compared to WSe_2_ (Figure S14b), reflecting the fact that the spin–orbit coupling in WS_2_ (397 meV) is appreciably smaller than that in WSe_2_ (476 meV).^48^ Another notable observation from Figure 5b is the presence of multiple polaritonic states (indicated by *) for WSSe (*x*_Se_ = 0.42) nanotubes. Figure 5c displays the variation of the polaritonic peaks with composition. The energies of the α and β peaks decrease with increasing Se content much like the A exciton (Figure 5d), while the energy of the γ peak is composition independent. Figure 5d presents the energy of the polaritonic α peak and the pure exciton A peak as a function of *x*_Se_. The energy of the polariton is lower compared with the exciton transition, which is a manifestation of the strong coupling effect.

### DFT Computational Results

**Figure 6:**
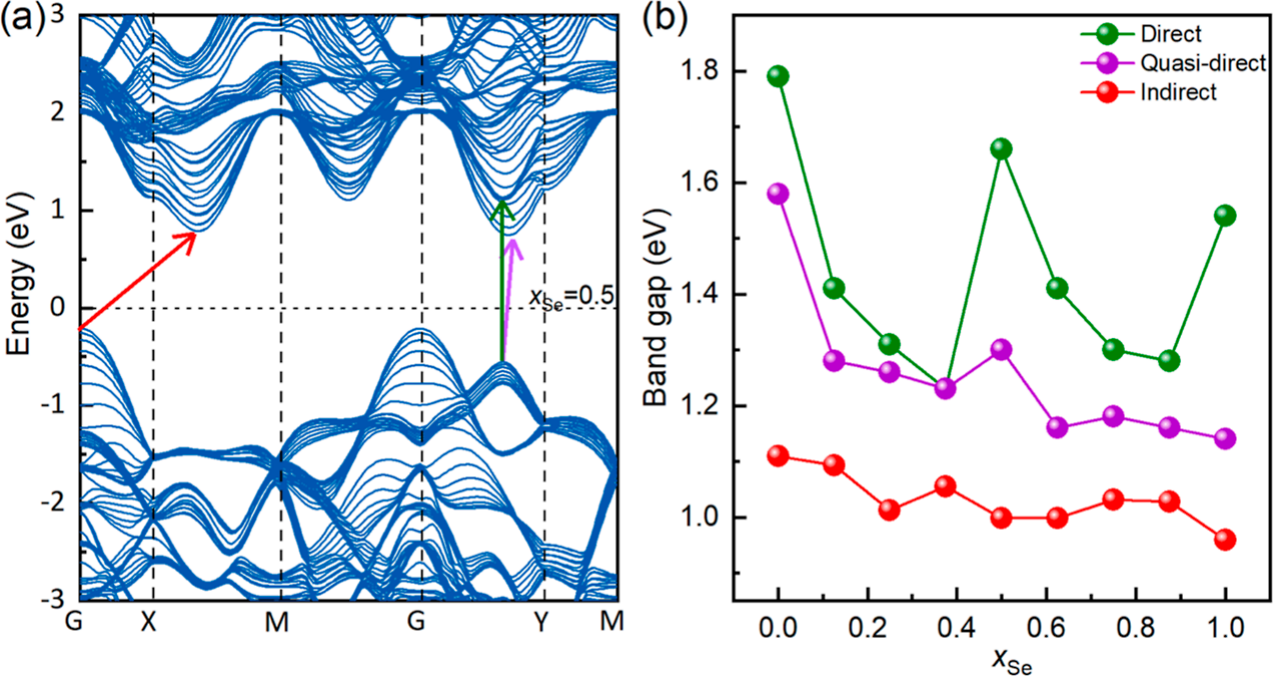
(a) Calculated band structures of WSSe, *x*_Se_ = 0.5 (see Supporting Information, Figure S15, for different *x*_Se_), where the high-symmetry points of the first Brillouin zone include *G* (0, 0, 0), *X* (0.5, 0, 0), *M* (0.5, 0.5, 0), and *Y* (0.0, 0.5, 0). The Fermi level is set as zero. The inserted red, dark green, and purple arrows represent the indirect, direct, and quasi-direct transitions, respectively. (b) Direct, quasi-direct, and indirect transition band gaps as a function of Se content in WS_2(1–*x*)_Se_2*x*_.

To elucidate the impact of the composition on the band gap of WS_2(1–*x*)_Se_2*x*_, band structure calculations were carried out for nine compositions (from *x* = 0 to *x* = 1 in steps of 0.125, Figure S15 and Table S4). For each of the compositions, the band structure was calculated for a slab of eight layers that are periodic in the 2D plane. Figure 6a shows the calculated band structure of a WSSe (*x* = 0.5) eight-layer slab. The indirect, direct, and quasi-direct band gap transitions are indicated (see also Table S4). One can note from this figure that there is a very small difference in the position of the direct and quasi-direct transition at the conduction band edge. However, being the lowest direct transition of the two, the quasi-direct gap is believed to better represent the measured values of the A exciton in the experiments (Figure 6b). From this figure, one notes that the calculated band gap shows a trend of overall decline following an increase in the selenium content. A slightly increased gap is observed at *x* = 0.5, probably due to the high symmetry of the multilayer structure. Notwithstanding the difference between an eight-layer slab and multiwall nanotubes, the DFT calculations and experiments show similar trends for the variations of the band gaps. The change in the band gap does not vary linearly with the composition. The reduction of the band gap at *x*_Se_ < 0.4 is quite significant compared to the reduction after *x*_Se_ ≥ 0.4.

### FDTD Simulations

To elucidate the nature of the strong light–matter interactions, FDTD simulations were performed. Figure S16a,b summarizes the FDTD simulations for WS_2_^38,39^ and WSe_2_ nanotubes, respectively. The solid black lines represent the photonic cavity modes inside the nanotubes. It is to be noted that the development of the photonic modes in pure WSe_2_ nanotubes is possible in smaller tube diameters compared to WS_2_ tubes due to the high refractive index of the materials in comparison to the pure WS_2_ nanotubes. The clear red-shift of the extinction peaks with respect to the A excitons in the absorption spectrum of WSe_2_ nanotubes and the dip are the hallmark of the strong-coupling effect.^38,39^Table S5 summarizes the peak positions for all types of studied nanotubes.

The FDTD calculations for WS_2_ nanotubes have been discussed before^38,39^ and will be only briefly mentioned here. In essence, the calculations show that the lowest energy polariton results from hybridization of the (TEM_01_) cavity mode with the A exciton for tubes with diameters 80 nm and above. The simultaneous interaction of the two lowest cavity modes (TEM_01_) and (TEM_02_) with the A and B excitons (630 and 520 nm, respectively) lead to an asymmetric peak (middle polariton) between the two dips of the extinction at 630 and 520 nm. Also, in agreement with the experimental data, the FDTD calculations showed (ref (38)) that the strong coupling is manifested clearly in the extinction spectrum (absorption + scattering) and is appreciably weaker in the net absorption mode. Furthermore, a strong Rabi splitting of 280 meV was calculated for 100 nm WS_2_ tubes.^39^ The refractive index of the materials can be tuned by controlling the Se content of the nanotubes. Due to the high refractive index of Se-rich materials, the experimental data (see Figure 5a) shows that the first photonic cavity mode (TEM_01_) can couple to tubes as narrow as 60 nm compared to Se-poor tubes (80 nm and above). Hence, the strong coupling between excitons and cavity modes is expected to increase with increasing Se content. The experimental observation is confirmed by the FDTD calculations showing that strong coupling occurs for WSe_2_ tubes 60 nm in diameter and above, leading to a low-energy polariton with a peak of around 820 nm (1.51 eV). As seen from Figure S16, the picture is much more complex for higher order cavity modes and higher energy polaritons. First, due to the higher refractive index of WSe_2_ compared to WS_2_, the second cavity mode (TEM_02_) couples with the A exicton for nanotubes with diameter 100 nm in wavelengths 780 nm and below. Simultaneously, the higher polariton of the first (TEM_01_) cavity mode and the lowest polariton derived from the coupling of the (TEM_01_) cavity mode with the B-exciton lead to a series of convoluted peaks between 600 and 750 nm for WSe_2_ tubes. Note also that due to the high absorption coefficient in this range, the cavity modes are damped. The calculated Rabi splitting for WSe_2_ nanotubes 80 nm in diameter is ≈140 meV. Therefore, the results of the FDTD calculations seem to be consistent with the recorded extinction spectra of WS_2_ and WSe_2_ tubes.

### Transient Absorption

TA pump–probe experiments were found to be very informative for elucidating the strong-coupling effect in such nanotubes.^39,49,50^ WS_2_ nanotubes with (average) diameter of 95 nm showed a very unique dynamics of the excited states. The lowest energy absorption dip Δ*A*/*A* at 670 nm (equivalent to the lowest peak of the transmission peak—Δ*T*/*T*) exhibited a considerable blue-shift of 30 nm within the first 100 ps, while nanotubes with an average diameter of 35 nm showed a very small shift (∼7 nm) of the first dip (640 nm) at this time interval.^39^ The dynamics of the exciton–polariton coupling in WS_2_ nanobutes^49^ and fullerene-like nanoparticles^50^ has been studied recently. In the first study,^49^ a phenomenological coupled oscillator model was established to analyze the data. The model considered coupling between an optical cavity mode and two absorption resonances A and B. It included effects such as band gap renormalization and trion formation and concluded a decay time of 8.3 ps for the polariton. In the second work, the time-dependent transformation of the exciton and cavity modes into polaritons was discussed via a different kinetic model.^50^ Here, a time constant of about 2 ps was found for the exciton-cavity mode coupling to form the polaritons. Furthermore, a decay time as long as 100 ps was calculated for the lowest polaritons (α). This long coupling time is a manifestation of the strong coupling effect and the Rabi splitting.

**Figure 7:**
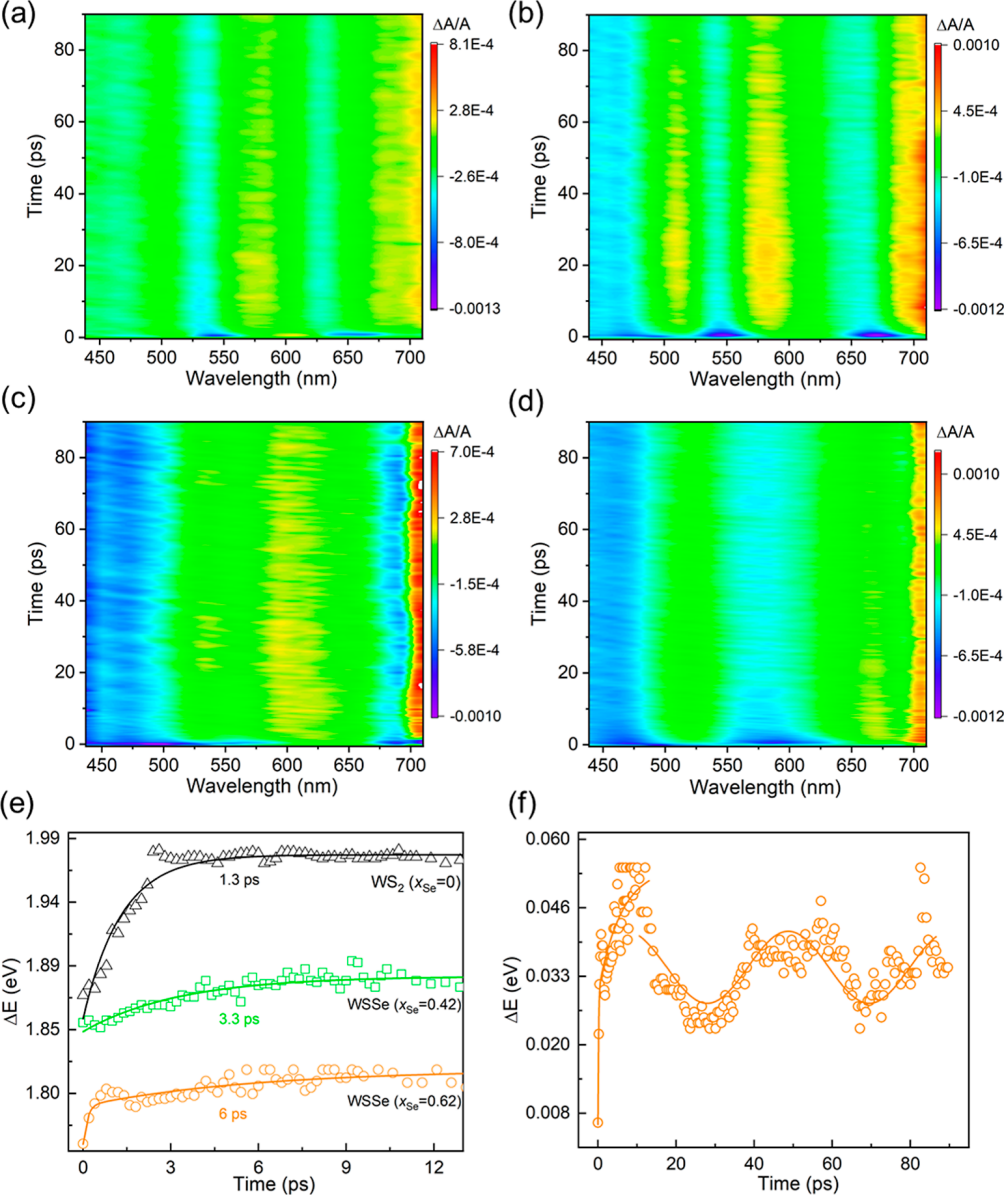
TA (Δ*A*/*A*) profile of (a) WS_2_, (b) WSSe (*x*_Se_ = 0.42), (c) WSSe (*x*_Se_ = 0.62), and (d) WSe_2_ nanotubes (up to 10 and 90 ps delay). Note the oscillating signal ∼690 nm for WSSe (*x*_Se_ = 0.42) nanotubes, which is not present in other materials. (e) Temporal dynamics of exciton–polariton transitions in the ultrafast time scale for WS_2_, WSSe (*x*_Se_ = 0.42), and WSSe (*x*_Se_ = 0.62) nanotubes. (f) Oscillating dynamics of incoherent to coherent transition in WSSe (*x*_Se_ = 0.62) nanotubes.

It is believed that the relatively weak light fluence (0.65 mJ/cm^2^) used in the present experiments excludes such mechanisms as band gap renormalization and trion formation, as discussed in great detail in ref (49). Figure 7a–d shows the TA-time map for WS_2_, WSSe (*x*_Se_ = 0.42), WSSe (*x*_Se_ = 0.62), and WSe_2_ nanotubes, respectively, in the first 90 ps. Figure S17 shows the dynamics of the TA in the first 9 ps. The TA of pure WS_2_ tubes synthesized by the present method (average diameter of 80 nm) was not much different from the TA of 80 nm diameter WS_2_ nanotubes reported before^39^ for both time scales; see Figure S17a. Due to the detection limit of the TA detector at 710 nm, the TA of the first extinction peak (A) of the last two Se-rich samples (Figure 7c,d) is not available. The similar TA dynamics profiles for the WSSe nanotubes with the composition *x*_Se_ = 0.18 and 0.77 for the time delay 9 and 90 ps are shown in Figure S18.

To better understand the dynamics of the distinct optical modes of the nanotubes in the different time domains, it is necessary to realize the TA spectra at short time scale (just after the pump, ∼300 fs) and the long time scale (∼1–3 ns). In analogy to ref (50), Figure S19 shows the wavelength derivative of the absolute absorption (d*T*_A_/dλ) and the short time TA spectra (red dotted line) for nanotubes with different Se contents. The nice resemblances between the TA spectra after a short delay time and the derivative of absolute absorbance imply that primarily the effect of the pump is associated with pure absorbance of the materials in ultrafast time scale (power absorbed by excitons). Likewise, Figure S20 represents the wavelength derivative of the extinction measurements (d*T*_E_/dλ) and the TA spectra at long delay time (red line) for the same kinds of nanotubes. These results imply that at long time delays, the spectral features can be ascribed to the coupled states (polaritonic states). Thus, it can be concluded that in the ultrafast TA, a short time lapse is required to couple the exciton with the cavity mode forming polaritons in the nanotubes. The delay times for the polaritons formation are 10–100 times slower than the lifetime of the pure exciton (few ps). It is to be noted that the choice of the short and long delay time (1–3 ns) is considered by the visual agreement between the derivative spectra of the net absorption and extinction with the TA signal, respectively.

The dynamics of the excitons and polaritons with different Se contents in the nanotubes are displayed in Figure 7. For the nanotubes of all compositions, the large energy shift of the dips (blue) and peaks (yellow-red) within the first 5–10 ps implies the formation of polaritonic states due to the strong coupling of excitons and trapped cavity modes. In all the materials, an exponential energy shift of the bleached state (first dip) (blue trace in the long wavelength in Figure 7) is observed within the time window of 5–10 ps following the excitation.^50^ The exciton–polariton decay rate constant is much slower for the Se-rich nanotubes compared with pure WS_2_ nanotubes. This is probably due to the strong photonic modes available close to the excitonic states (the high refractive index of WSe_2_ implies efficient photon trapping). Figure 7e shows the temporal dynamics of the energy shift (the first dip) for WS_2_, WSSe (*x*_Se_ = 0.42), and WSSe (*x*_Se_ = 0.62) nanotubes. Slowing down of the calculated time constant is observed with increasing Se content of the nanotubes. This slowdown is probably correlated with the increase in the refraction index of the nanotubes with increasing Se content,^51^ which induces slower light propagation in the media.

The temporal energy shift (exciton–polariton transition) in the TA for all different compositions of Se including WS_2_ nanotubes shows a uniform decay within 15 ps and onward. However, an exception is observed for WSSe nanotubes with *x*_Se_ = 0.62 (Figure 7f). Here, following the early exponential decay, oscillatory behavior of the lowest TA mode (peak) at delay times of a few tens ps is observed (Figure 7f). The calculated frequency period of this oscillatory mode is ∼48.1 GHz. At longer delay times, a low frequency osciallation of this mode (peak) with a frequency of 446.23 MHz is observed for the WSSe (*x*_Se_ = 0.62) nanotubes (Figure S21). These oscillations are believed to be a manifestation of the coherent wave propagating in these nanotubes. The slow oscillation periods could be also related to the large refractive index of the nanotubes and slow light propagation in the Se-rich phase.^51^

## Conclusions

Nanotubes of ternary alloys WS_2(1–*x*)_Se_2*x*_ in the entire composition range (0 ≤ *x* ≤ 1) were prepared via solid vapor reaction in a sealed ampoule. WO_2.72_ nanowhiskers with definite sizes were pre-prepared and served as sacrificial templates, and hence, the produced nanotube shows a uniform size (diameter) distribution. The reaction proceeds through Kirkendall diffusion (chalcogen atoms in and oxygen out); however, the reactivity of sulfur and selenium differed appreciably. Sulfur was found to be more truculent with respect to the oxide whiskers compared to selenium. Structural characterization showed that the lattice of the WSSe nanotubes is strongly modulated with composition. Atomic resolution electron microscopy images and chemical maps revealed that both sulfur and selenium coordinated in a trigonal prismatic fashion to tungsten and distributed rather randomly in the anion lattice sites. Low-loss EELS shows that the band gap shrinks from 1.95 to 1.5 eV upon full substitution of sulfur by selenium. Hints exist for a deviation of the band gap reduction from linear regime as a function of composition with a minimum (1.2 eV) near *x*_Se_ = 0.6–0.7. The band structure calculations of eight-layer WS_2(1–*x*)_Se_2*x*_ slabs with varying *x*_Se_ endorse the observed deviation. The quasi-direct gap in the *k* point of the Brillouin zone was found to shrink from 1.6 to 1.14 eV upon sulfur to selenium substitution. The WSSe nanotubes exhibit strong light–matter interactions, where the cavity of the nanotubes acts as Fabry–Perot interferometers. The peaks of the extinction spectra show clear red-shift compared to the excitonic transitions of the net absorption, which was attributed to a strong coupling effect resulting in polariton quasiparticles. FDTD calculations revealed that owing to the large refractive index (∼5), WSe_2_ nanotubes with diameter as small as 60 nm could confine the light and form polaritons, whereas for WS_2_ nanotubes, the cavity modes appear at >80 nm diameter tubes. TA pump–probe studies corroborated the extinction and absorption results and strong coupling effects. It is seen that at very short time scales, the transient extinction spectrum is purely excitonic in nature and polaritonic signatures appear after a short delay. These findings confirm the notion that the cavity modes couple with the excitons and form polariton quasiparticles. The lifetime of these states increases substantially upon enrichment of the nanotubes with selenium atoms.

Therefore, ternary alloys of transition metal dichalcogenide nanotubes offer a new way to tune the optical, electrical, and chemical properties in 1D nanostructures with numerous potential applications, as indicated by this research.

## Experimental Section

### Synthesis of WS_2(1–*x*)_Se_2*x*_ (0 ≤ *x* ≤ 1) Nanotubes

The nanotubes of various compositions of WS_2(1–*x*)_Se_2*x*_ were prepared in a sealed quartz ampoule using a two-zone vertical furnace fitted with one end closed quartz tube. In each experiment, 25 mg WO_2.72_ nanowhiskers and stoichiometric amounts of sulfur and selenium (see Table S1, Sigma-Aldrich 99.99%) along with 4–5 mg of NaBH_4_ were weighed separately in a N_2_ filled glovebox and transferred into a quartz ampoule. The sodium borohydride is unstable above 400 °C and releases hydrogen, which provides the necessary reducing atmosphere. The WO_*x*_ whiskers were filled in a small quartz cup and placed 15 cm away from the S/Se powder inside the ampoule (see Figure 1c). This separation was necessary because the experiments carried out by mixing WO_*x*_ whiskers with S/Se led to the collapse of the nanowhiskers and did not yield any nanotubes. The ampoule with all the precursors was transferred safely without exposing to moisture and connected to a vacuum system equipped with a diffusion pump, which was backed by a rotary vacuum pump. The system was pumped down to 10^–5^ to 10^–6^ Torr, and the ampoule was sealed using a torch flame. The sealed ampoules were subjected to an annealing process using two different temperature zones in the furnace (Figure 1c). The bottom edge of the sealed ampoule with S/Se and NaBH_4_ was kept in zone 1 and was ramped to 450 °C at the rate of 3 °C/min and then to 840 °C by ramping at a rate of 5 °C/min. The whiskers were kept in zone 2 and were ramped directly to 840 °C at 5 °C/min. Figure S2 shows the temperature–time profiles of the synthesis chosen for these experiments.

The WO_*x*_ whiskers were allowed to react with S/Se and H_2_ at this temperature for 3 h. At the end of the reaction, the ampoule was cooled at a rate of 3 °C/min. The dark blue WO_*x*_ powder in the quartz cup was converted by the chalcogenide vapors into black powder, confirming that the reaction was completed and the WS_2(1–*x*)_Se_2*x*_ or pure WSe_2_ powders were obtained. In the case of pure WS_2_ nanotubes, the whiskers were inserted into a preheated furnace and allowed to react for 30 min and the ampoule was retracted outside the hot zone and cooled to room temperature naturally. The product powders were further used for various characterization. Note that the mole fraction of the sulfur and selenium atoms in the precursor, expressed through *X*_Se_ and *X*_s_, is generally quite different from the true composition in the nanotubes (*x*_Se_ and *x*_s_) determined via EDS.

## Computational Details

### DFT Simulations and Model Structures

The DFT calculations, including the geometry optimizations and electronic structure calculations, were carried out by the Vienna Ab Initio Simulation Package (VASP) code with the generalized gradient approximation within the Perdew–Burke–Ernzerhof functional.^52^ The construction and post-processing of calculation results were performed using the atomistic simulation environment.^53^ The interaction between the core and valence electrons was described by the projected augmented wave pseudopotential.^54^ The valence electron wave functions were expanded by the plane-wave basis set with kinetic-energy cutoff of 500 eV. The 11 × 11 × 1 *k*-point samplings of the first Brillouin zone were used for geometry optimization and electronic properties calculations for unit cell of 2D sheets, respectively. The DFT-D3 correction of Grimme method was adopted to correct the interlayer van der Waals interaction.^55^ All structures (lattice parameters and atomic positions) were completely relaxed until the forces were less than 0.05 eV/Å and energy tolerances were less than 1.0 × 10^–5^ eV, respectively. The multilayer WS_2_, WSe_2_, and WS_2(1–*x*)_Se_2*x*_ with *x* = 0, 0.125, 0.25, 0.375, 0.5, 0.625, 0.75, 0.875, and 1 are constructed by stacking the orthorhombic unit cell of WS_2_ in an AB patterns. To avoid a randomization of the S–Se distribution, we replace S with Se in each layer one by one. A vacuum space of 20 Å perpendicular to the plane of 2D nanosheet was considered to avoid self-interaction by the boundary conditions.

## Supporting Information Available

Experimental and characterization details; computation details; XRD, HR-STEM, and STEM–EDS analyses; Raman spectroscopy; DFT band structure of WSSe layers; steady-state optical extinction and absorption; pump–probe optical measurement-TA spectroscopy; FDTD simulations; and absorption and extinction derivative plots (PDF)
