# Supplementary material for: Nanotubes from Ternary WS2(1–x)Se2x Alloys: Stoichiometry Modulated Tunable Optical Properties
Source: J Am Chem Soc. 2022 Jun 3;144(23):10530–42. doi: 10.1021/jacs.2c03187 (PMC9204813; doi:10.1021/jacs.2c03187)
Supplement: Supplementary file 1 — ja2c03187_si_001.pdf [file ja2c03187_si_001.pdf]

Supporting information for
Nanotubes from Ternary $WS_{2(1-x)}Se_{2x}$ Alloys: Stoichiometry Modulated Tunable Optical Properties

M. B. Sreedhara,[1] Yana Miroshnikov,[1] Kai Zheng,[2] Lothar Houben,[3] Simon Hettler,[4,5] Raul Arenal,[4,5,6] Iddo Pinkas,[3] Sudarson S. Sinha,[7*] Ivano E. Castelli[2*] and Reshef Tenne[1*]

[1]Department of Molecular Chemistry and Materials Science, Weizmann Institute of Science, Rehovot 7610001, Israel
[2]Department of Energy Conversion and Storage, Technical University of Denmark, DK-2800 Kgs. Lyngby, Denmark
[3]Department of Chemical Research Support, Weizmann Institute, Rehovot 7610001, Israel
[4]Instituto de Nanociencia y Materiales de Aragon (INMA), CSIC-Universidad de Zaragoza, 50018 Zaragoza, Spain
[5]Laboratorio de Microscopias Avanzadas (LMA), Universidad de Zaragoza, 50018 Zaragoza, Spain
[6]ARAID Foundation, 50018 Zaragoza, Spain
[7]Department of Chemistry, Physics and Atmospheric Sciences, Jackson State University, Jackson, Mississippi 39217, United States

[*]corresponding authors emails: Sudarson S. Sinha: sudarson.s.sinha@jsums.edu; Ivano E. Castelli: ivca@dtu.dk and Reshef Tenne: reshef.tenne@weizmann.ac.il

SI Content

1. *Synthesis of $WO_{2.72}$ nanowhiskers*
2. Characterization details
   2.1. X-Ray diffraction
   2.2. SEM And SEM-EDS
   2.3. TEM, STEM and EELS
   2.4. Raman Spectroscopy
   2.5. Steady state optical extinction and absorption
   2.6. Pump-Probe Optical Measurements-Transient absorption spectroscopy
3. Derivation of absorption and extinction derivative
4. FDTD simulation details
5. DFT calculations
6. SI Figures: S1-S21
7. SI References

1. Synthesis of $WO_{2.72}$ nanowhiskers:
Fast growth of long suboxide ($W_{18}O_{49}$= $WO_{2.72}$) nanowhiskers was accomplished through the following steps: reduction, sublimation, and condensation. For this synthesis, a precursor of $WO_{2.92}$ nanoparticles was used. The growth was carried out in a horizontal furnace using a quartz tube reactor under a slightly reducing atmosphere.

In a typical synthesis, the precursor powder was placed into a quartz boat. The reactor was purged continuously with nitrogen gas. The total gas-flow rate was about 100- 150 cm^3/min^{-1}. The flow rate of the hydrogen was varied slightly between 0.5 and 5 cm^3 min^{-1} with the rest being nitrogen carrier gas. The boat was pushed into the heated zone of a horizontal furnace, which was

preheated to 780 - 840 ℃. The reactants were maintained at this temperature for 15-30 min. Once the reaction terminated, the boat was withdrawn from the oven and left to cool down naturally to room temperature under the flow of nitrogen gas. The best product for the next step was obtained at the following parameters: about 0.5% H_2 in the reaction gas and relatively low temperature (780 °C) for 15 min. As prepared whiskers were characterized by XRD and SEM and were subsequently used for the synthesis $WS_{2(1-x)}Se_{2x}$ ($0 \leq x \leq 1$) nanotubes. The whiskers were 25-100 nm in diameter and half to a few mμ long. **Fig. S1**a in the supporting information (SI) shows a scanning electron microscope (SEM) image of the oxide nanowhiskers, while **Fig. S1**b presents the X-ray powder diffraction (XRD) pattern of such oxide nanowhiskers (space group *P*2/*m* *a*=1.83, *b*=0.38 and *c*=1.4 nm).[1]

2. Characterization details

<u>*2.1. Scanning electron microscopy (SEM) and Energy dispersive X-ray spectroscopy (EDS)*</u>:

Scanning electron microscopy (SEM) analysis was done with a Zeiss Sigma 500 model. A minute quantity of native sample was picked up by a capillary tube and dispersed on carbon tape for the SEM analysis. Energy dispersive X-ray spectroscopy (EDS) analysis and mapping were performed using a retractable quadrants detector (Bruker QUANTAX FlatQUAD), The quantification of the elements is based on a standard-less and self-calibrating spectrum analysis procedure, using the ZAF matrix correction formulas. The statistical distribution of the size of the nanotubes was estimated by analyzing many SEM images of the product.

<u>*2.2. X-ray powder diffraction*</u>:

X-ray powder diffraction (XRD) was performed using TTRAX III (Rigaku, Tokyo, Japan) theta-theta diffractometer. The set-up was equipped with a rotating copper anode X-ray tube operating at 50 kV/200 mA. The samples were prepared on a zero-background Si substrate. They were scanned using the Cu Kα line in specular diffraction mode ($\vartheta/2\vartheta$ scans) from 4-90° (2ϑ) with a step size of 0.02° and a scan rate of 0.5° per min.

<u>*2.3. Raman spectroscopy*</u>:

A minute quantity of the sample was dispersed in ethanol by sonication and drop casted on a glass substrate. Raman scattering measurements in the range from 100 to 1000 cm^{-1} were recorded on individual NTs in back-scattering geometry. A LabRAM HR Evolution spectrometer (HORIBA, France) equipped with different lasers was employed. For the 633 nm laser, the maximum incident power on the sample was 0.225 mW. Given the spot size (1 µm) of the laser, the real incident power on the NT was ~ 0.02 mW. The LabRAM is fitted with an 800 mm spectrograph with a very high spectral resolution and low stray light. Frequency calibration was performed before every measurement session using the Si peak at 520.7 cm^{-1} of single-crystalline Si(100). The measurements were recorded with a 600 groves/mm grating with ∼1.8 cm^{-1} pixel resolution. The NTs were illuminated using several microscope objectives (MPlanFL NA=0.9, Olympus, Japan). The system utilizes an open confocal microscope (Olympus BXFM) with a spatial resolution better than 1 µm. Due to the very high aspect ratio of the NTs, it was very easy to visualize an individual nanotube with 100x/150x objective and analyze it. The Raman spectra were collected in a 1024 × 256 pixel front illuminated CCD camera (Syncerity, HORIBA, USA) with open electrode which was cooled to -60 °C.

<u>*2.4. Transmission electron microscopy (TEM), STEM-EDS and EEL Spectroscopy*</u>:

Regular transmission electron microscopy (TEM) and selected area electron diffraction (SAED) patterns analyses were performed using a JEOL JEM2100 microscope operated at 200 kV, a Talos

F200X G2 TEM 200 kV (Thermo Fisher Scientific, USA), and an image-corrected Titan[3] (Thermo Fisher Scientific, USA) microscope operated at 300 keV. The analysis of the TEM images, including intensity profiles along the *c*-axis, and the SAED has been performed with Digital Micrograph 3.1.0 (Gatan) software and ImageJ.

A double hexapole aberration-corrected Themis Z microscope (Thermo Fisher, USA) equipped with a high-brightness FEG was employed for HRSTEM imaging and monochromated EEL spectroscopy at an accelerating voltage of 200 kV. HAADF-STEM images were recorded with a Fischione Model 3000 detector with a semi-convergence angle of 21.4 mrad, a probe current of 40 pA, and an inner collection angle of 70 mrad. EDS hyperspectral maps were collected with a SuperX G2 four-segment SDD detector with a probe semi-convergence angle of 21.4 mrad, a beam current of approximately 200 pA, a pixel dwell time of 10-20 µs and a total recording time of typically 10 minutes. Quantitative maps were analyzed with the Velox software (Thermo Fisher, USA), through background subtraction, spectrum deconvolution and quantification based on theoretical cross-section data. A correction of frame-to-frame beam/specimen drift was employed where required using custom software in order to refine net intensity profiles. Monochromated EEL spectra were recorded at a system energy resolution of 80 meV on a Gatan Quantum GIF 966ERS energy loss spectrometer (Gatan Inc., Pleasanton, USA) equipped with an Ultrascan1000 CCD camera. The EEL spectra were recorded with a STEM probe with a semi-convergence angle of 24 mrad and a beam current of 200 pA by summing multiple 2 ms spectrum acquisitions from a spectrum image map taken over a larger field of view to distribute the electron exposure. In all cases of atomic-resolution HRSTEM analyses prior specimen cleaning steps, e.g. plasma cleaning, were avoided in order to preserve the surface structure of the nanostructures.

EELS measurements for bulk plasmon analyses have been performed in an image-corrected Titan[3] (Thermo Fisher Scientific, USA) operated at 300 keV. EELS data was acquired with a Gatan Image Filter Tridiem at an energy dispersion of 0.05 eV/channel and an acceptance angle of 11.9 mrad. The bulk plasmon energy E_P was determined by fitting the energy-loss function in dependence of the energy loss E.[2]

$$Im\left[\frac{-1}{\varepsilon(\omega)}\right] = \frac{E \cdot \Delta E_P \cdot E_P^2}{\left(E^2 - E_P^2\right)^2 + (E \cdot \Delta E_P)^2}$$

with $\varepsilon(\omega)$ the dielectric function and ΔE_P the plasmon energy width. Prior to fitting, the background from the zero-loss tail (power-law function) was subtracted from the spectra.

2.5. Steady-state extinction and absorption measurements:
UV–Vis extinction spectra were measured using a Cary-5000 UV-VIS-NIR spectrometer (Varian). In all the cases, a few milligrams of samples were dispersed in spectroscopic grade ethanol and ultra-sonicated for a few minutes to get the uniform dispersions. The samples were quickly dispersible and the suspension was stable during measurement. All suspensions were measured in quartz cuvettes in the range of 1000 to 400 nm with 1 nm interval.

Integrating sphere (Hamamatsu Quantaurus QY) was used to measure the absolute absorbance of the dispersed $WS_{2(1-x)}Se_{2x}$ (0≤x≤1) nanotubes. The samples are placed inside an integrating sphere for the measurements. This instrument directly measures the amount of absorbed light in the sample, by placing the sample in the integrating sphere. The system was calibrated each time using a reference sample with known absorbance (quartz cuvette with pure ethanol to extract the

net optical absorbance. A calibration for counting the single-pass absorption photons was performed to avoid the full extinction which also includes photons that are scattered a few times before reaching the detector.

2.6. Pump-Probe Optical Measurements:

The transient absorption setup used for this study consisted of a 420 nm ultrashort pulse (roughly 120 fs pulse duration) of light produced by an amplified Ti:Sapphire system (Spitfire ACE, Mai Tai SP, Empower 45, Spectra-Physics, Santa Clara, CA) coupled to an OPA (TOPAS-Prime, and NIRUVIS harmonic generator, Light Conversion, Vilnius, Lithuania). Following the pump pulse, the sample was probed at varying delays by a white-light continuum from a CaF_2 window (with a spectral range of 370–750 nm (a filter placed in the beam path to reduce the intensity of the fundamental beam (800 nm)), covering the visible-near infrared (vis–NIR) region. The pump and probe beams cross and overlap within the cuvette holding the sample and the probe beam is collected into an optical fiber bringing it into a Triax 190 spectrograph (Horiba Jobin Yvon, France). The spectrograph is equipped with a Newton CCD camera (Andor, UK), which collects every pulse of the 1 kHz train of probe pulses from the laser system and calculates an absorption spectrum from two consecutive pulses of probe light (one with and one without the pump pulse which is removed by a synchronized mechanical chopper). The system utilizes delay lines (Aerotech, USA) for the pump and probe beams fitted with retroreflectors, allowing for delays of up to 4 ns, which is controlled by LabView software. The data was analyzed using SurfaceXplorer (Ultrafast Systems, Sarasota, FL). Transients were acquired with sub-picosecond resolution up to 4 ns.

3. Derivation of absorption and extinction derivative

The transmission % can be expressed in terms of absorbance as

$$\%T(\lambda) = 10^{-A(\lambda)} \qquad (1)$$

Taking derivative with respect to the wavelength (λ) in both side of equation (1),

$$\frac{dT(\lambda)}{d\lambda} = -2.303\, T(\lambda)\frac{dA(\lambda)}{d\lambda} \qquad (2)$$

Hence,

$$-\frac{1}{T(\lambda)}\frac{dT(\lambda)}{d\lambda} = 2.303\,\frac{dA(\lambda)}{d\lambda} \qquad (3)$$

According to Beer-Lambert law:

$$A(\lambda) = \varepsilon_A(\lambda)\, C\, l \ldots\ldots\ldots\ldots\ldots\ldots\ldots\ldots\ldots\ldots(4)$$

Where, A, ε_A C, l are absorbance, absorption coefficient, concentration, and optical path length respectively. The absorbance and the extinction coefficient are frequency dependent.

Using Eq. (4) in Eq. (3),

$$-\frac{1}{T_A(\lambda)}\frac{dT_A(\lambda)}{d\lambda} = 2.303\, C\, l\frac{d\varepsilon_A(\lambda)}{d\lambda} \qquad (5)$$

where $T_A(\lambda)$, $\varepsilon_A(\lambda)$ are the transmission and absorption coefficients in the absorption experiment, respectively. In a similar way, one can also express the extinction measurement in terms of the extinction coefficient (ε_E) as

$$-\frac{1}{T_E(\lambda)}\frac{dT_E(\lambda)}{d\lambda} = 2.303\, C\, l\frac{d\varepsilon_E(\lambda)}{d\lambda} \qquad (6)$$

Where $T_E(\lambda)$, $\varepsilon_E(\lambda)$ are the transmission and extinction coefficient in absorption experiment, respectively.

4. Finite-difference time-domain (FDTD) Simulations

FDTD simulations were carried out using a commercial Lumerical FDTD software, where Maxwell's equations are solved numerically in space and time. As the length of the nanotubes is considerably larger compared to the relevant wavelengths, it is possible to get meaningful results while using 2D simulation, i.e. considering the cross section of the nanotubes, only. Here, the nanotubes are simulated as infinitely long cylinders with diameter span from 10 to 200 nm with 2 nm resolution and surrounded by an environment with a constant refractive index similar to the solvent (n_0 = 1.3).

To simulate the optical response of the semiconducting nanotubes, the dielectric function of bulk WS_2 and WSe_2 was used.[3] To reproduce pure cavity modes without the contribution of the excitonic features, additional simulation was performed with a constant refractive index (n = 4.0 for WS_2 and 5.0 for WSe_2). For both cases, a broadband plane wave source (unpolarized) was used to illuminate the nanotube. Two separate monitors were placed to record the absorbed and scattered electromagnetic field. Data from these monitors were used to calculate the normalized extinction cross-sections. Note that the normalization was performed with respect to the highest recorded signal. To minimize the calculation time, symmetry conditions were considered for the transverse magnetic (TM) modes (the field is parallel to the long axis of the nanotube) and antisymmetric conditions for the transverse electric (TE) modes (the field is perpendicular to the nanotube long axis). The polarization of the electric field of the source was considered parallel and perpendicular to the long axis of the nanotube. A schematic representation of the simulation setup is similar to the one used in ref. 48 of main text.

5. DFT calculations:

It is noted that there are quasi-direct gaps in multilayer $WS_{2(1-x)}Se_{2x}$ compositions, as represented by the purple lines in **Fig. S14**, which shifted slightly along with the k-vector in conductive bands compare with the direct band gaps between high-symmetry points of G (0, 0, 0) and Y (0.0, 0.5, 0) and show smaller gaps for optical transitions, thus the quasi-direct gaps are more favorable than direct band gaps for optical transitions in multilayer $WS_{2(1-x)}Se_{2x}$ compositions.

5. SI Figures: S1-S14

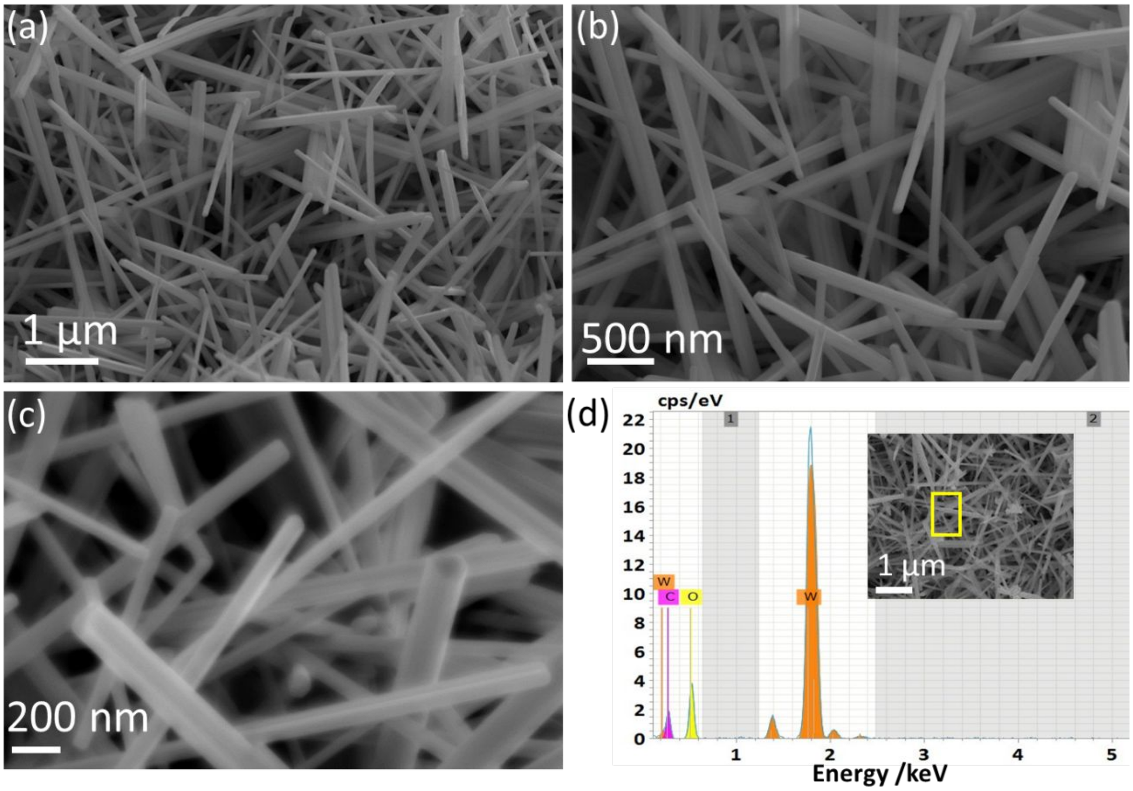

Fig. S1. (a-c) Low and high magnification SEM micrograph of $WO_{2.72}$ nanowhiskers, the average size of the whiskers was found to be around 100 nm. (d) SEM-EDS spectrum of the nanowhiskers, SEM image in the inset shows the region from which EDS spectrum collected. The semi-quantitative analysis shows that the concentration of oxygen and tungsten approximately match the stoichiometric $WO_{2.72}$.

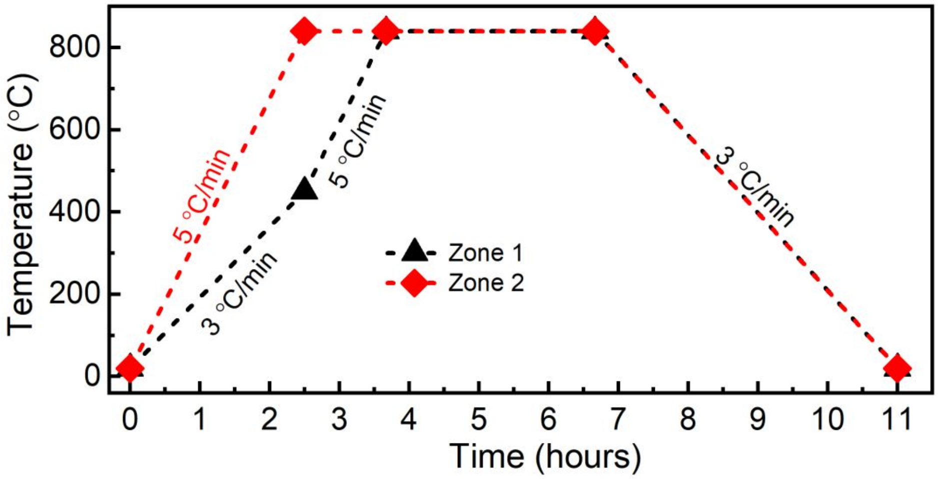

Fig. S2. The temperature profile of the two-zone vertical furnace during the nanotube synthesis as a function of time. At the beginning zone-2 of the furnace was ramped quite fast (5 °C/min) compared to

zone-1 (3 ℃/min) in order to achieve faster sulfurization/selenization rate (i.e. to reduce the competition between sulfurization/selenization and the reduction reactions).

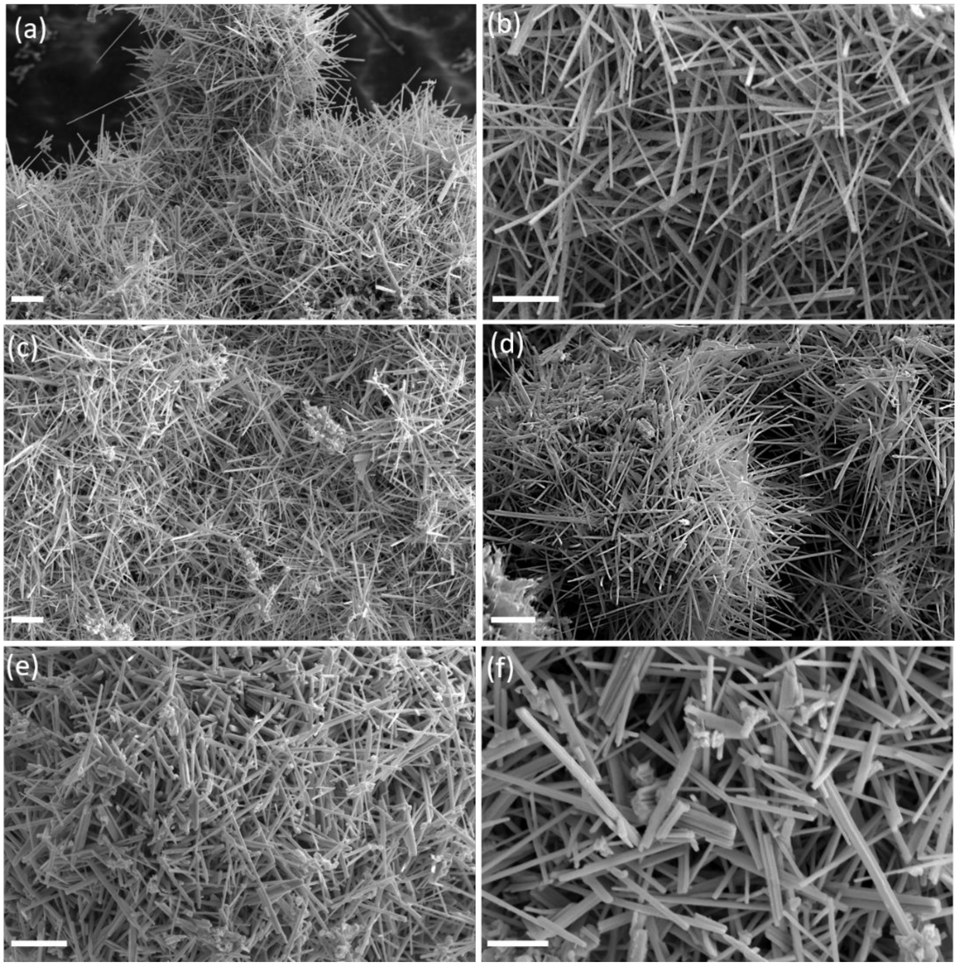

Fig. S3. SEM micrographs of $WS_{2(1-x)}Se_{2x}$ nanotubes (a) WSe_2 x_{Se}=1, (b) WSSe, x_{Se}=0.77, (c) WSSe, x_{Se}=0.62, (d) WSSe x_{Se}=0.42, (e) WSSe x_{Se}=0.18 and (f) WS_2, x_{Se}=0. Scale bars (a-e) 2 µm and (f) 1 µm, respectively.

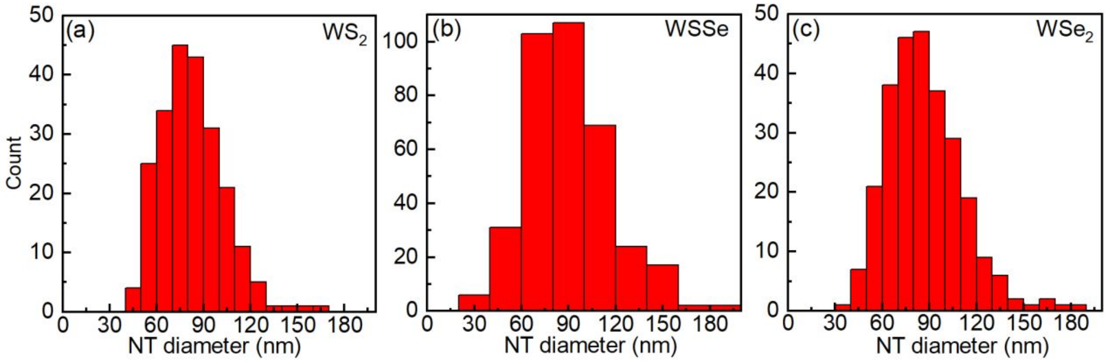

Fig. S4. The size distribution of (a) WS_2, (b) WSSe (x_{Se}=0.42) and (c) WSe_2 nanotubes analyzed based on several SEM images of the nanotubes.

Table S1. Description of the few reactions carried out in the present project. Influence of the sulfur and selenium content, reaction time and temperature on the products and their morphologies produced during synthesis. In all cases reported $WO_{2.72}$ whiskers from the same batch were used as a precursor with 3-4 mg of $NaBH_4$.

X_{Se}	X_S	reaction time (h)	reaction temperature (°C)	reaction products	observed morphology
1.0	0.0	3.0	840	WSe_2 (majority) +WO_2	nanotubes+ whiskers with the surface covered with WS_2
0.90	0.10			$W(S_{0.25}Se_{0.75})_2$	nanotubes
0.75	0.25			$W(S_{0.38}Se_{0.62})_2$	nanotubes
0.50	0.50			$W(S_{0.58}Se_{0.42})_2$	nanotubes
0.25	0.75			$W(S_{0.82}Se_{0.18})_2$	nanotubes
0.0	1.0			WS_2	flakes with triangular geometry
0.0	1.0	3.0	700	WS_2+WO_2 (major)	Whiskers of oxide with the surface covered with WS_2
		#3.0	840	WS_2	flakes with triangular geometry
		*1.0	840	WS_2 + WO_2	nanotubes with the surface covered with flakes
		*0.5	840	WS_2+WO_2 (major)	nanotubes + whiskers with the surface covered with WS_2
1.0	0.0	3.0	700	WSe_2+ WO_2 (major)	Oxide whiskers (core) with surface covered with few-layers of WSe_2 (shell)

#reaction carried out without using $NaBH_4$.
*indicates that ampoules with precursor were inserted directly to the hot zone and retracted from the hot zone (natural cooling) after the reaction.

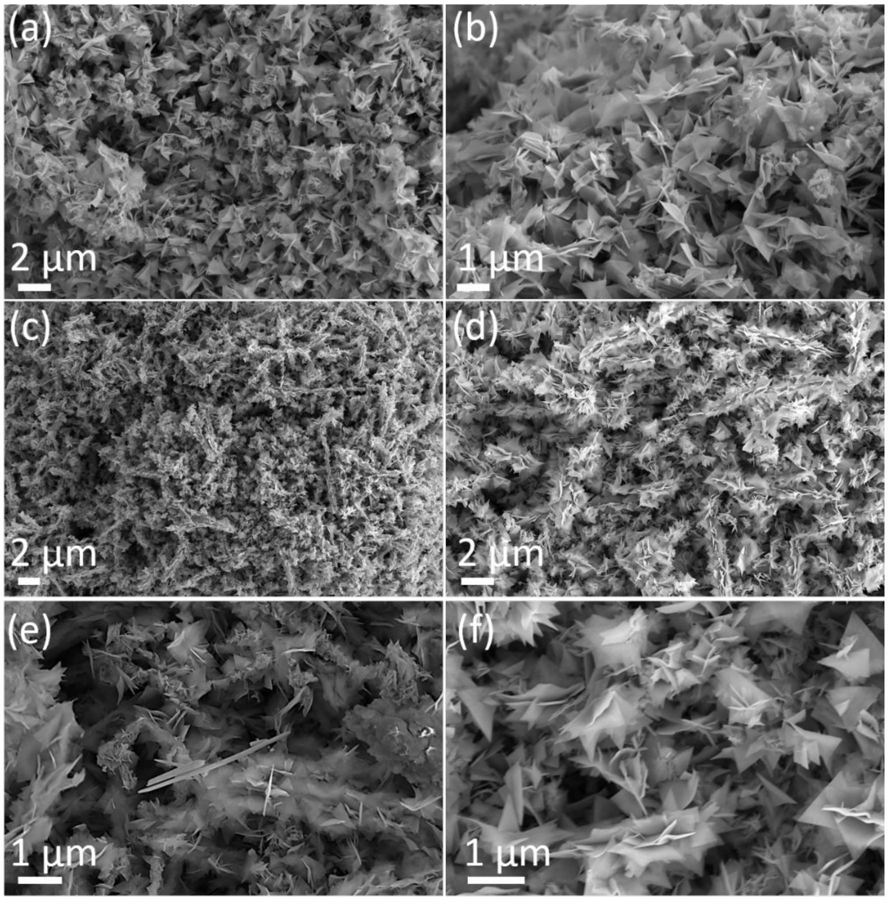

Fig. S5. SEM micrographs of WS_2 products obtained during the optimization process for the WS_2 nanotubes synthesis. WS_2 flakes obtained during the reaction process of 3 hours (a-b) and one hour (c-f) with regular heating and cooling processes (according to temperature profile). The reaction of sulfur in the absence of $NaBH_4$ also yielded the flakes.

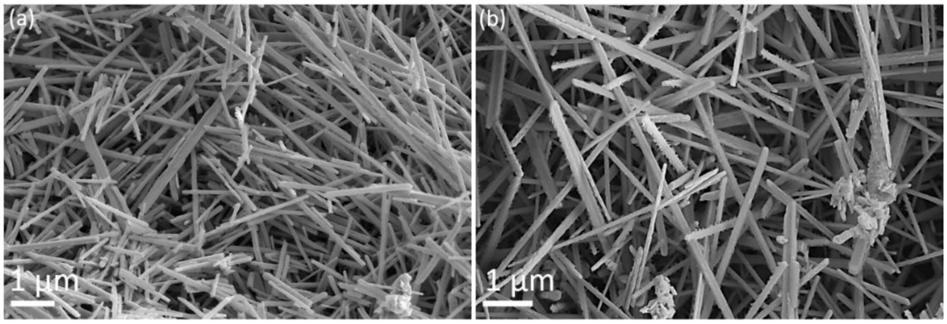

Fig. S6. SEM micrographs of reaction products obtained upon inserting precursors directly into the hot zone of the pre-heated furnace and retrieved after one hour. The flakes are already started growing on the surface of the whiskers and is clearly visible in (b). The reaction stopped at 30 mins yielded nanotubes of WS_2 (see **Fig. S3**f).

Table S2. Description of the nominal and observed concentrations of sulfur and selenium (in at%) used for synthesis and in the nanotubes, respectively. The corresponding nominal and observed stoichiometries were presented. X_S, X_{Se} are the nominal concentrations and y_S and y_{Se} are the observed (average) concentrations obtained by EDS analysis. For simplicity all the compositions were referred with simple term x_{Se}, i.e. $x_{Se}=y_{Se}/(y_S+y_{Se})$. The quantification was done via STEM-EDS (of individual nanotubes) and the average values have been taken. The results of the SEM-EDS analysis are similar to the ones presented here.

Compound $WS_{2(1-x)}Se_{2x}$	X_S in (%)	X_{Se} in (%)	y_S in (%)	y_{Se} in (%)	Observed stoichiometry $x_{Se}=y_{Se}/(y_S+y_{Se})$
X=1, (WSe_2)	-	100	-	100	x_{Se}=1, (WSe_2)
X=0.9, ($WS_{0.2}Se_{1.8}$)	10	90	23	77	x_{Se}=0.77, ($WS_{0.46}Se_{1.54}$)
X=0.75, ($WS_{0.5}Se_{1.5}$)	25	75	38	62	x_{Se}=0.62, ($WS_{0.76}Se_{1.24}$)
X=0.5, ($WS_{1.0}Se_{1.0}$)	50	50	58	42	x_{Se}=0.42, ($WS_{1.16}Se_{0.94}$)
X=0.25, ($WS_{1.5}Se_{0.5}$)	75	25	82	18	x_{Se}=0.18, ($WS_{1.64}Se_{0.36}$)
X=0, (WS_2)	100	-	100	-	x_{Se}=0 (WS_2)

X- nominal composition
X_S= nominal sulfur content; X_{Se}= nominal selenium content -used for the reaction
y_S= observed sulfur content, y_{Se}= observed selenium content –in the nanotubes
$x_{se}=y_{Se}/(y_S+y_{Se})$-observed composition (actual)

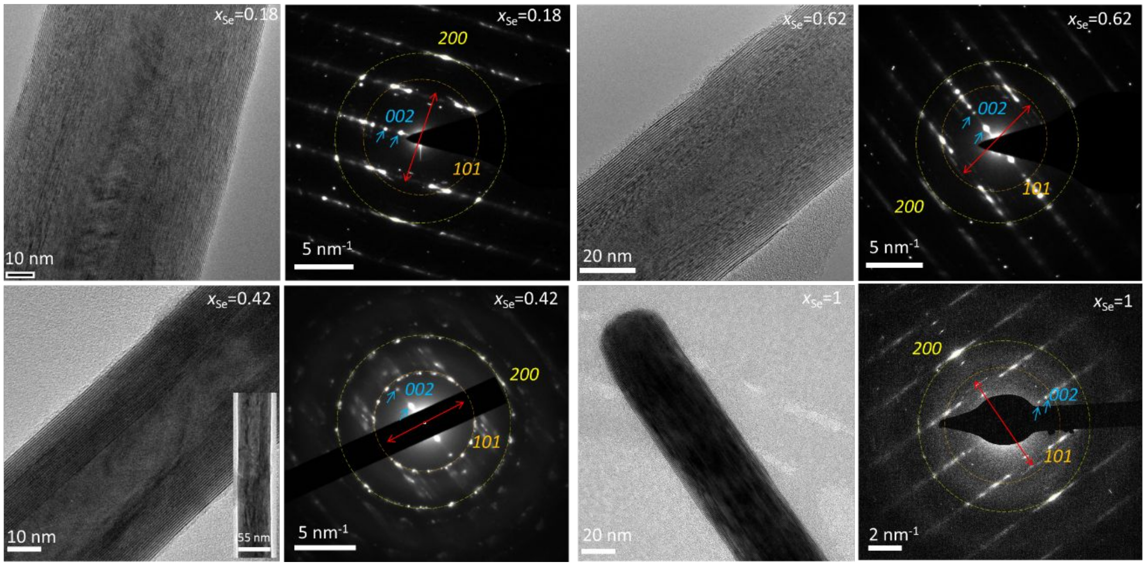

Fig. S7. HR-TEM Images and SAED patterns of WSSe nanotubes of different selenium composition, x_{Se}. Reflections corresponding to (002), (101) and (200) planes of the $W(S_{1-x}Se_x)_2$ lattice are marked by blue arrows, orange and yellow dashed circles, respectively.

Fig. S7 shows a TEM image along with the corresponding selected area electron diffraction pattern of a WSSe nanotubes of various compositions. The main *c*-axis reflections are marked by a blue arrow (d_{002} = 0.64 nm), green dashed circle (d_{101} = 0.27 nm) and orange dashed circle (d_{200} = 0.16 nm). These measured values correspond well to the ones determined by the XRD analyses. Six reflections appear for both *(101)* and (*200*) as expected for the hexagonal lattice, which indicates a single-crystalline structure in the investigated area. One set of *(200)* reflections (marked by dotted orange ellipses) coincides with this axis implying that the *a* axis of the hexagonal lattice

coincides with the nanotube axis. The nanotubes were found to exhibit small chiral angles. Analyses of additional SAED patterns show a single-diffraction pattern for the multiwall nanotube, i.e. all the nanotube walls show the same orientation. Only in few cases, the nanotubes exhibit multiple helicities, each one can be assigned to a different nanotube wall (e.g. x_{Se}=0.42). This latter case was predominantly evident for nanotubes with mixed S,Se composition. For all the nanotube exhibiting single diffraction pattern, the *a* axis coincided with the nanotube axis, indicating that this direction is favored during the growth. Presumably, this unique orientation is induced by the orientation of the WO_3 whiskers.

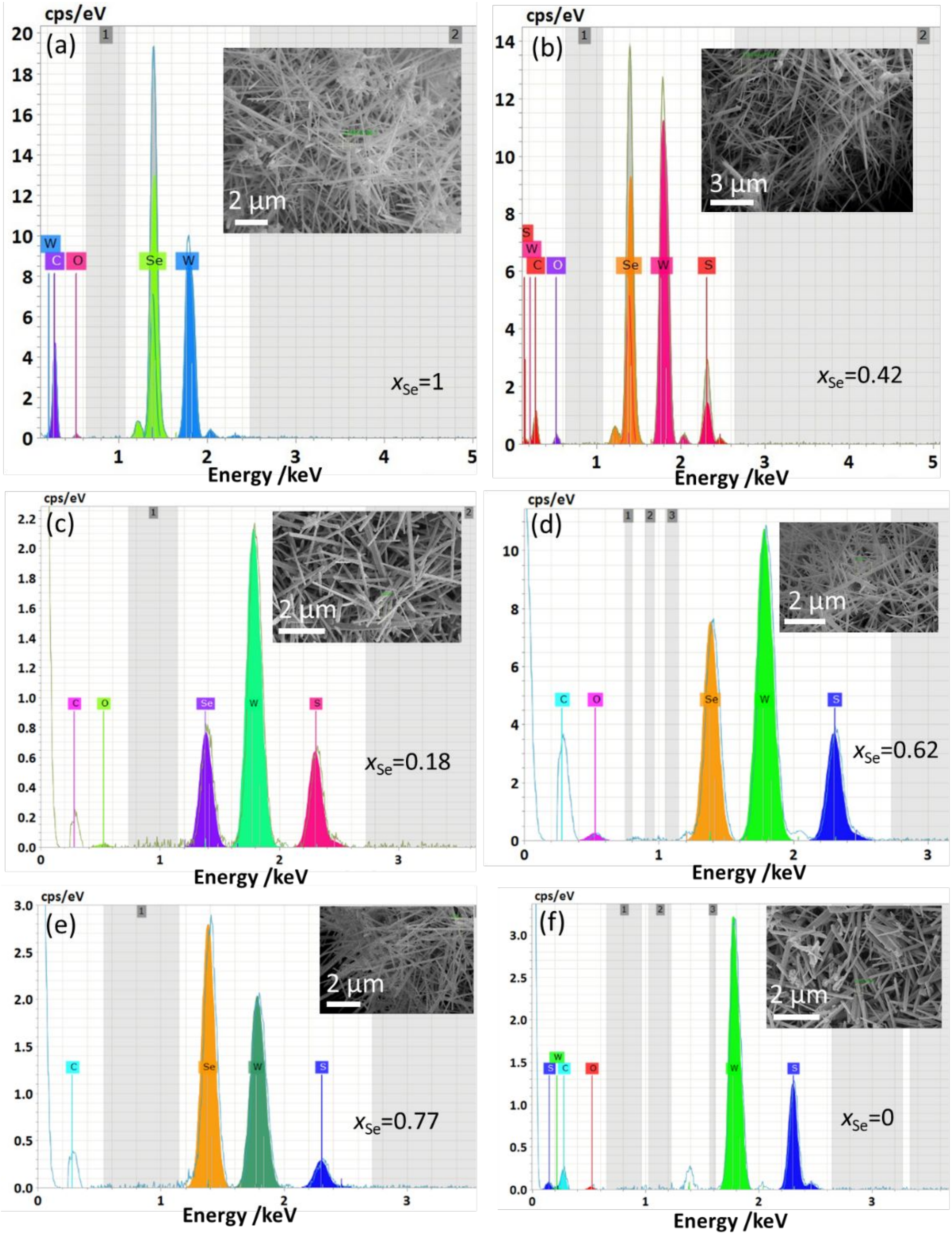

Fig. S8. (a-f) SEM-EDS analysis of WSSe nanotubes with varying composition of selenium, x_{Se}. Image in the inset shows the region where the EDS has been collected.

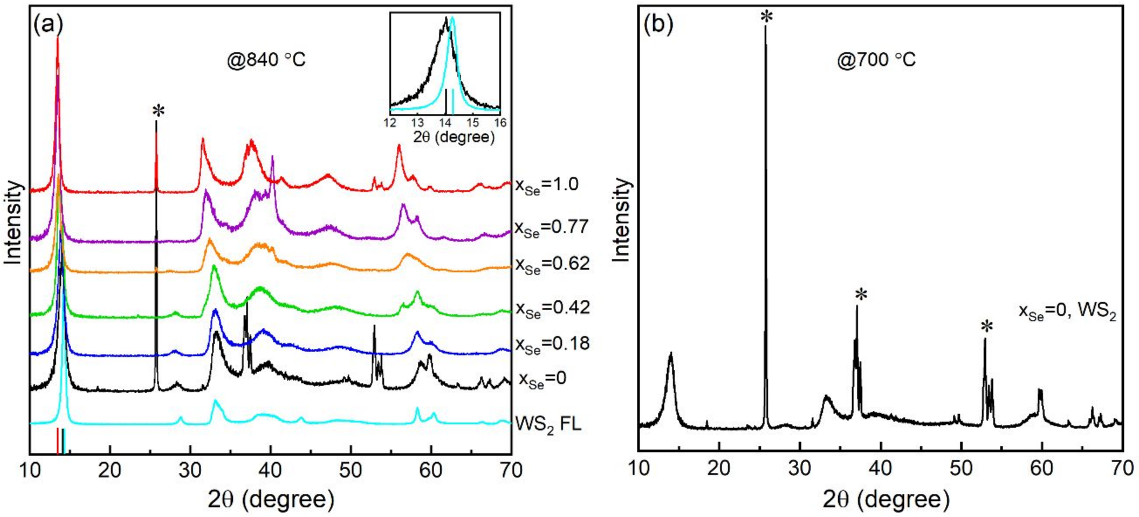

Fig. S9. XRD patterns of $WS_{2(1-x)}Se_{2x}$ ($0 \leq x \leq 1$) nanotubes in comparison with WS_2 nanoflakes, which were obtained from the $WO_{2.72}$ nanowhiskers. The position of the (*002*) Bragg plane corresponding to WS_2 flakes, WS_2 nanotubes and WSe_2 nanotubes is marked with cyan, black and red line, respectively. Extended view of the (002) plane of WS_2 flakes and nanotubes in the inset reveals that WS_2 layers in the nanotubes have larger interlayer spacing (*002*) compared to flakes, which is attributed to the well-known strain effect in the folded 1D structure. The Bragg peak marked with the * in the WS_2 and WSe_2 nanotubes patterns correspond to monoclinic WO_2 tungsten oxide (ICSD PDF-4 00-032-1393).[3] (b) XRD patterns of the product obtained relatively lower reaction temperatures (at 700 C) for WS_2 nanotubes. It is seen that the majority of the product to be WO_2 core with few-layers of WS_2 on the surface, indicating reduction reaction is more prominent.

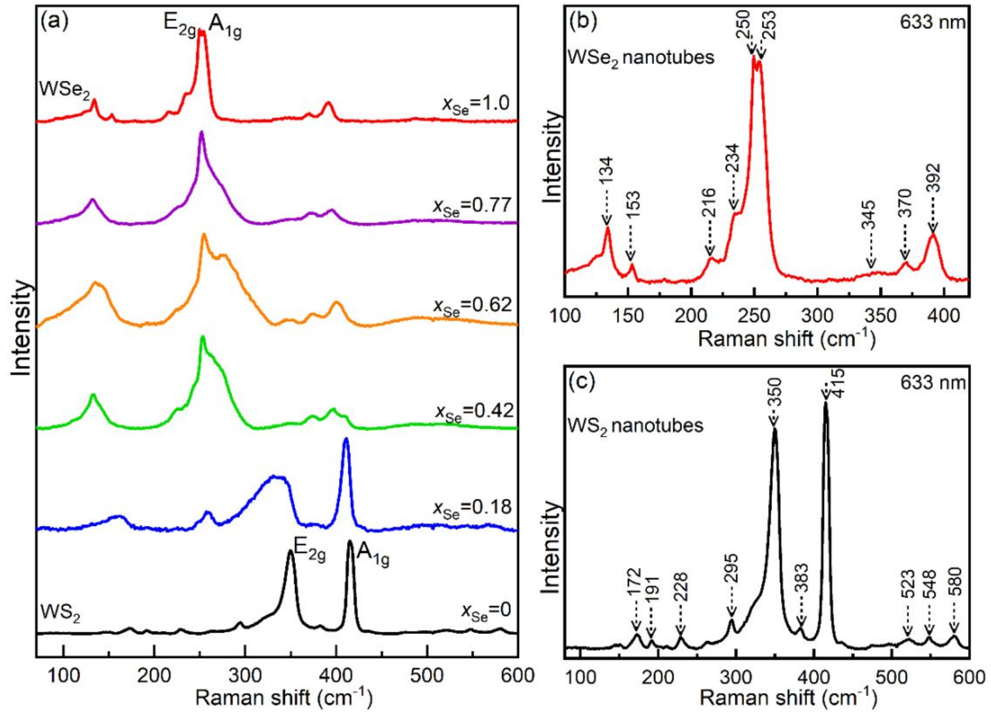

Fig. S10. Raman spectra of the $WS_{2(1-x)}Se_{2x}$ nanotubes for different x values. (a) evolution of the Raman spectra for nanotubes with varying selenium content (x_{Se}), (b) pure WSe_2 nanotubes and (c) WS_2 tube.

Table S3. Analysis of Raman modes of WSe_2 and WS_2 nanotubes recorded using 633 nm laser excitation.

WSe_2		WS_2	
Peak position	Raman mode	Peak position	Raman mode
133	A1g(M)-LA (M)	172	E_{2g}^{1}
215.4	E(K)	191	B_{2g}
233	E(M)	228	A_{1g}(M)-LA(M)
250 and 255 (de)	E_{2g} and A_{1g}	262	A_{1g}(M)-ZA(M)
≈345	-	295	2ZA(M)?
366	-	350	E_{2g}^{1}
387	-	383	2*LA*(*M*)- $2E_{2g}^{2}$(M)
		415	A_{1g}
		523	E_{2g}^{1}(M)+LA(M)
		548	

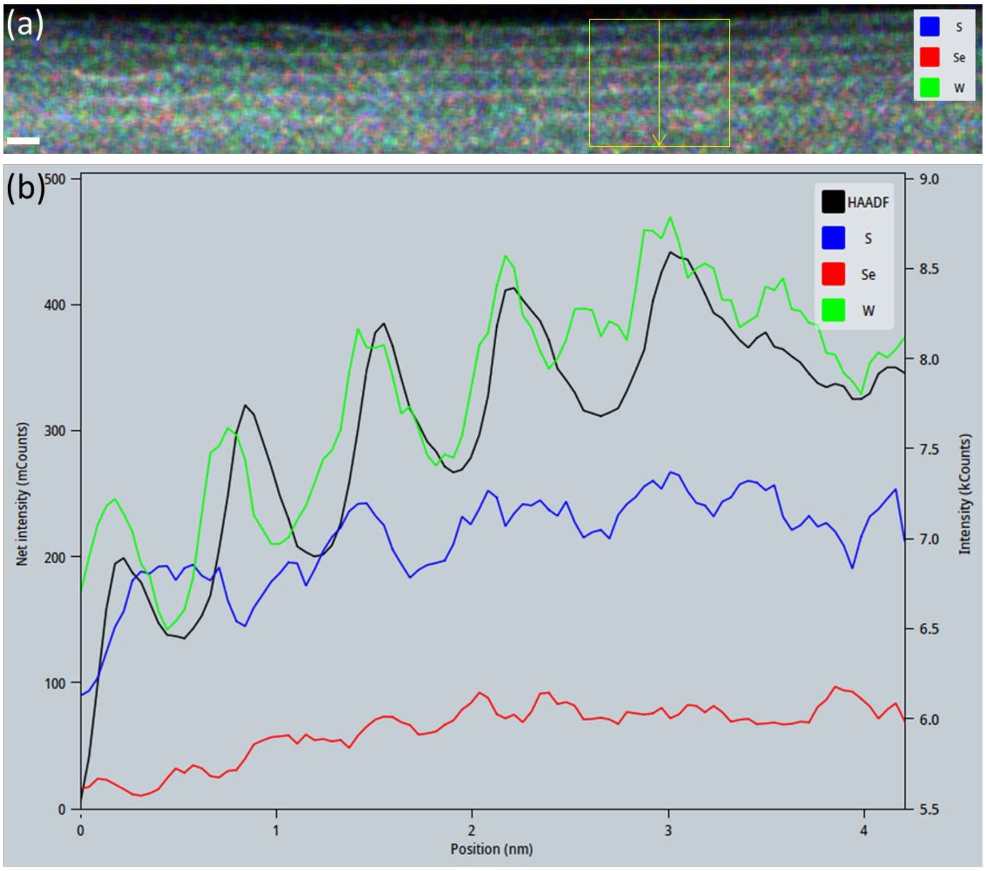

Fig. S11. (a) HRSTEM-EDS chemical mapping of top few-layers of WSSe nanotube with x_{Se}=0.42. (b) Net intensity profiles of chemical maps W, S and Se along with HAADF intensity derived from the region marked in the image (a).

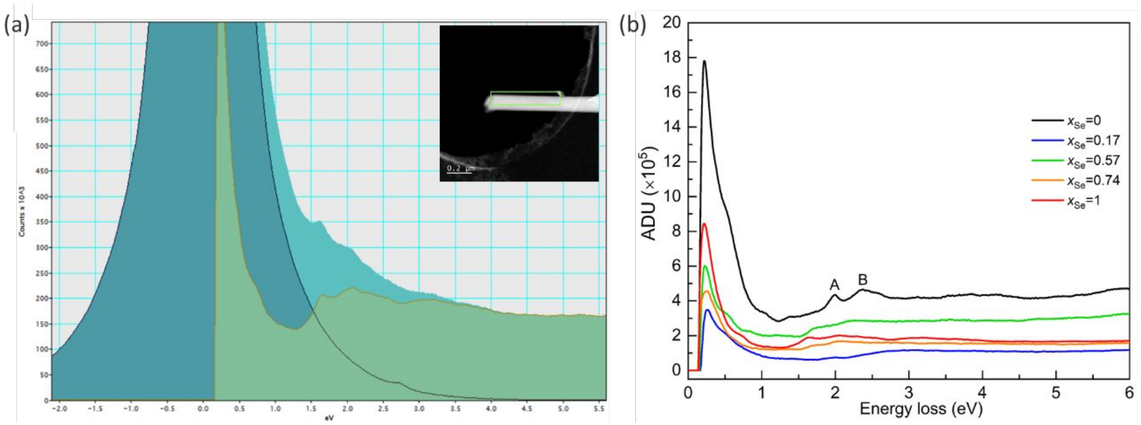

Fig. S12. Low-loss EEL spectra of WSSe nanotubes. (a) Low-loss spectrum of a WSe_2 nanotube showing the magnified part of extracted inelastic signal of low-loss EEL spectrum. The spectrum is integrated from a hyperspectral map collected from the region marked with a green rectangle in the annular dark-field image in the inset. Inter-band transitions are visible after subtraction of the reflected tail of the zero-loss peak. Residual tail intensity at energies below 1 eV originate from imperfect subtraction, phononic and plasmonic low loss transition. (b) Raw data for the elastic part of the low loss EEL signal for five different WSSe nanotube with varying selenium content, x_{Se}, after the subtraction of zero loss peak. The onset of interband transitions at an energy loss > 1.5 eV is evident, the absorption peaks related to A and B excitons are indicated.

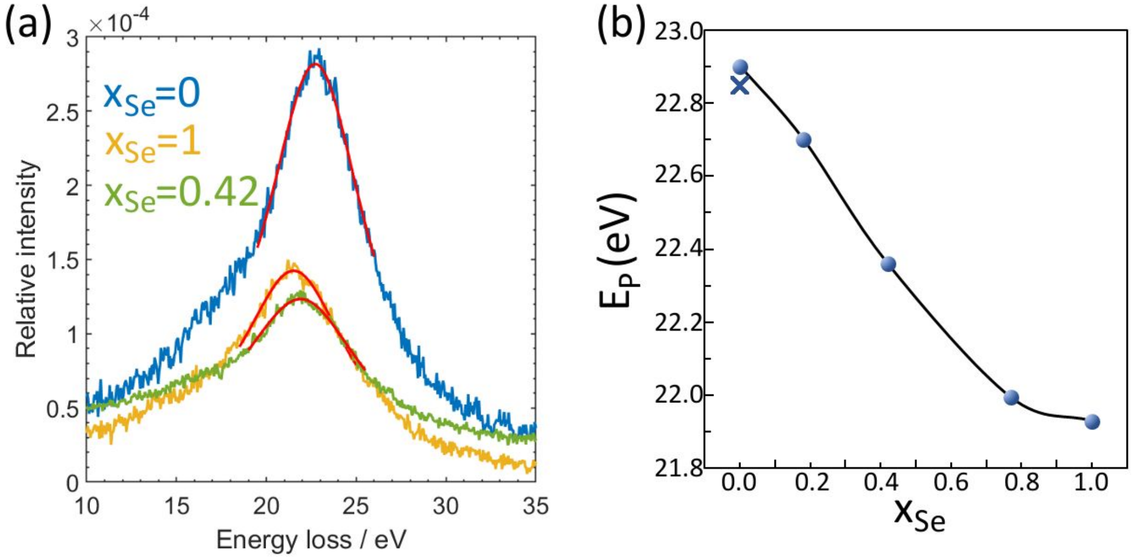

Fig. S13. (a) EEL spectrum of WSe, WSe_2 and WSSe nanotube of intermediate composition x_{Se}=0.42 around the bulk plasmon region. The bulk plasmon energy E_P (measured) decreases with increasing Se content. (b) Variation of the bulk plasmon energy of WSSe nanotubes as a function of selenium content. Plasmon energy obtained for WS_2 flakes is marked by a cross.

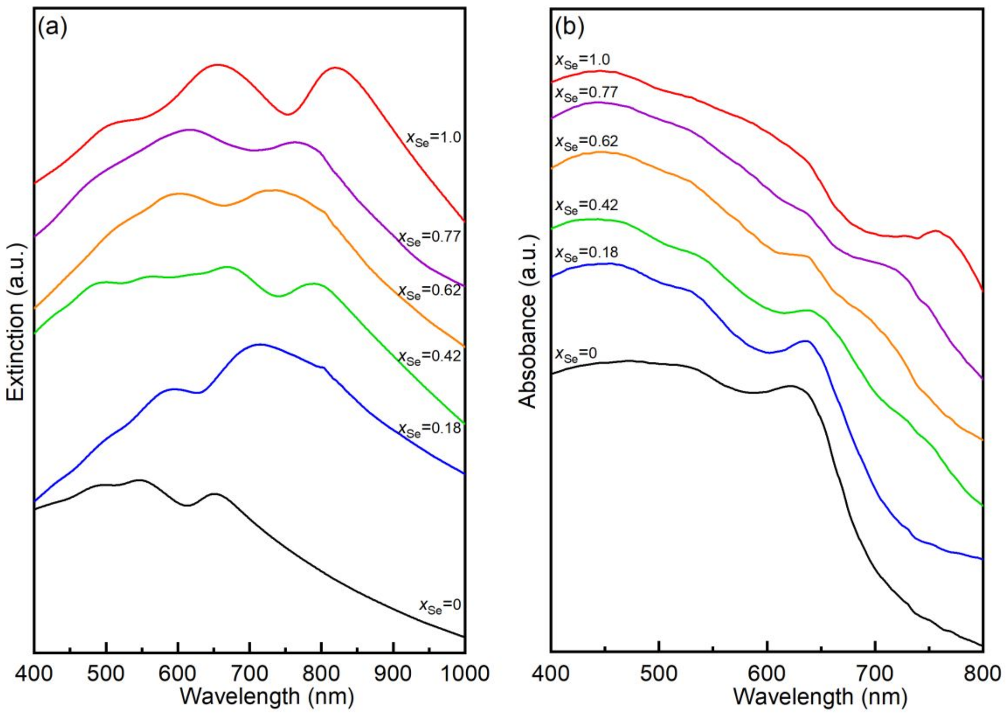

Fig. S14. Extinction (a) and Absolute absorption spectrum of WSSe nanotubes with varying Se composition, x_{Se}. Extinction spectrum is measured by simply placing the sample between the tunable light source and the detector. The net absorption spectrum is obtained by placing the sample in the center of an integrating sphere and irradiation with a tunable source.

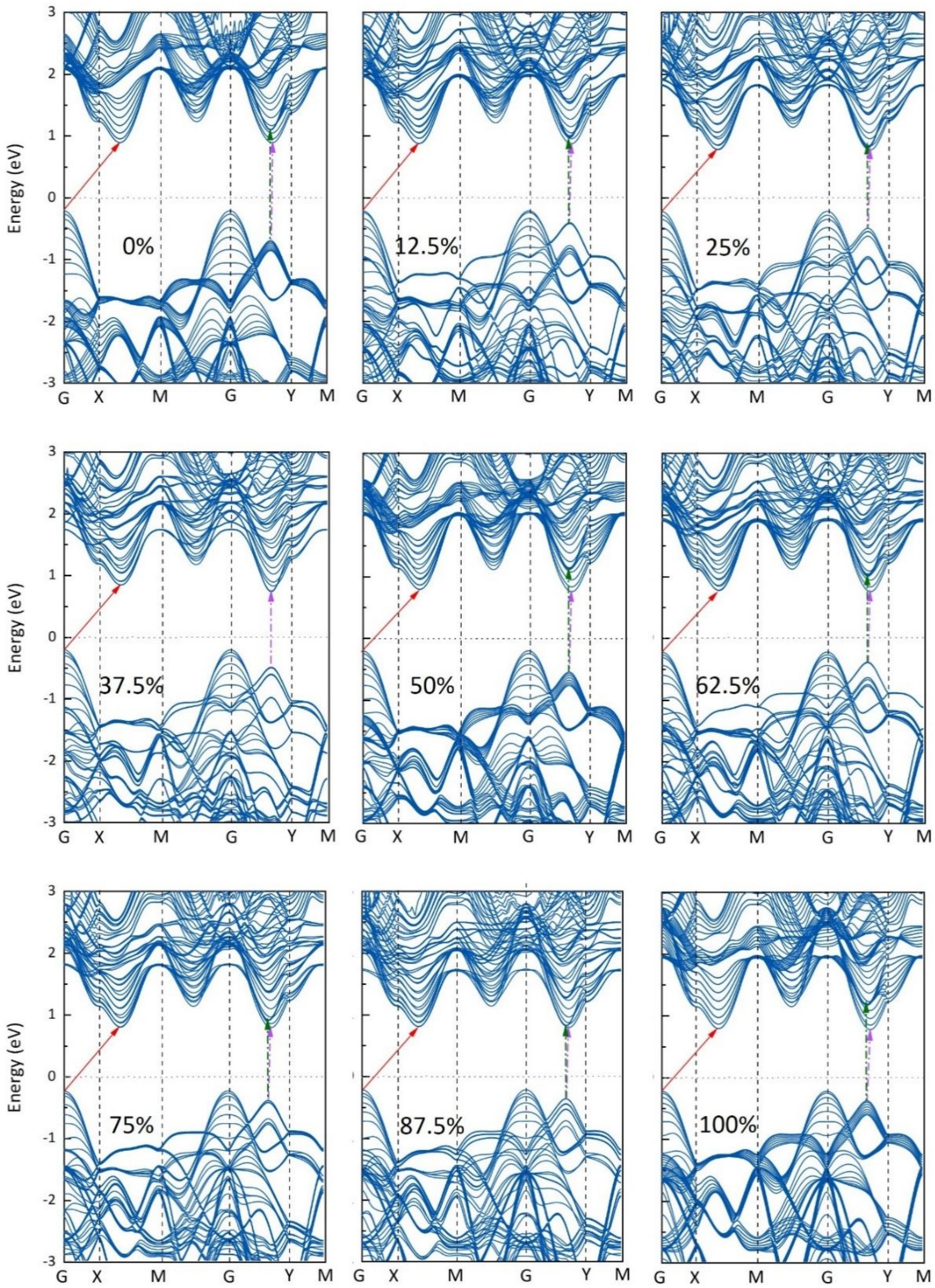

Fig. S15. The calculated band structures of different compositions with the increasing percent of Se in $WS_{2(1-x)}Se_{2x}$, where the high-symmetry points of the first Brillouin zone include G (0, 0, 0), X (0.5, 0, 0), M (0.5, 0.5, 0) and Y (0.0, 0.5, 0). The Fermi level is set as zero. The inserted red, dark green and purple arrows represents indirect, quasi-direct, indirect transitions, respectively.

Table S4. The calculated direct, quasi-direct, indirect band gaps (in eV) of $WS_{2(1-x)}Se_{2x}$ compositions with different Se contents.

Xse / Gaps	0%	12.5%	25%	37.5%	50%	62.5%	75%	87.5%	100%
Direct	1.79	1.41	1.31	1.23	1.66	1.41	1.3	1.28	1.54
Quasi-direct	1.58	1.28	1.26	1.23	1.30	1.16	1.18	1.16	1.14
Indirect	1.11	1.09	1.01	1.05	0.99	0.99	1.03	1.02	0.96

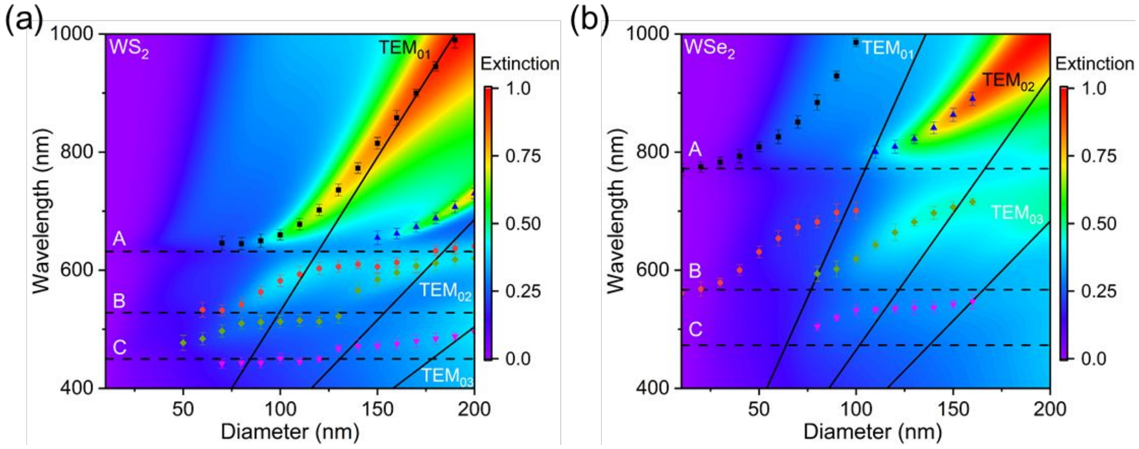

Fig. S16. FDTD simulations of the diameter dependence of the extinction of (a) WS_2 nanotubes; (b) WSe_2 nanotubes. The diagonal solid lines are the different cavity modes (TEM_{01}, TEM_{02}, TEM_{03}) persisting in the nanotubes. The horizontal dashed lines represent the different excitons in the materials. The symbols in the figures are the (calculated) maxima of the polaritonic modes for WS_2 and WSe_2 tubes as a function of the nanotube diameter. The black square- lowest polariton mode derived from the coupling of the (TEM_{01}) cavity mode with the A-exciton. However, the other peak positions (red circle, blue triangle, green diamond, magenta inverted triangle) are the result of convolution of higher-order cavity modes (TEM_{02}, TEM_{03}), the lower polariton of high energy exciton and upper polariton of low energy exciton. For details of the calculations see SI text.

***Table S5**: Summary of the spectral data calculated from the simulated results, presented in **Fig. S16**.*

Diameter (nm)	*Wavelength ±* Error *(nm)*	*Wavelength ±* Error *(nm)*	*Wavelength ±* Error *(nm)*	*Wavelength ±* Error *(nm)*	*Wavelength ±* Error *(nm)*
WSe_2 nanotubes					
10	770±10.2	561±9.5			
20	775±8.9	568±12.2			
30	783±8.49	579±7.3			
40	793±11.5	600±9.2			
50	809±8.3	631±10.1			
60	826±11.7	654±13.1			
70	851±10.8	673±13.3			
80	884±13.0	682±9.9	594±12.7	505±6.9	
90	929±8.0	698±14.4	602±13.3	520±5.6	
100	986±7.3	701±14.5	619±7.8	532±9.7	
110			643±7.5	533±4.5	800±10.5
120			664±14.0	535±8.1	809±10.9
130			682±7.3	536±11.3	822±7.6
140			697±10.8	537±10.2	841±9.9
150			707±9.5	542±7.0	863±11.2
160			716±5.8	547±12.1	890±11.2
WS_2 nanotubes					
50			477±12.8		
60		533±12.4	484±10.6		
70	646±12.0	532±9.3	497±10.6	442±8.1	
80	645±10.3	542±11.5	510±10.2	443±9.4	
90	650±11.2	563±9.4	512±11.3	443±8.2	
100	660±8.9	582±10.0	513±10.3	451±8.7	
110	678±9.6	593±13.1	515±9.9	445±5.9	
120	702±9.6	603±10.3	513±8.6	450±5.7	
130	736±10.0	606±9.7	522±11.3	467±5.8	
140	773±9.0	610±7.2	566±12.8	471±11.7	
150	815±9.8	606±10.9	584±10.2	472±7.1	655±11.5
160	858±12.6	613±8.7	596±10.1	475±10.1	662±8.8
170	900±6.4	607±10.9	607±13.4	480±10.6	673±8.5
180	945±8.0	633±7.7	612±13.1	484±9.2	688±9.5
190	990±13.9	637±9.8	618±10.6	488±6.3	707±10.2
200		641±11.3	620±12.4	495±7.7	730±8.8

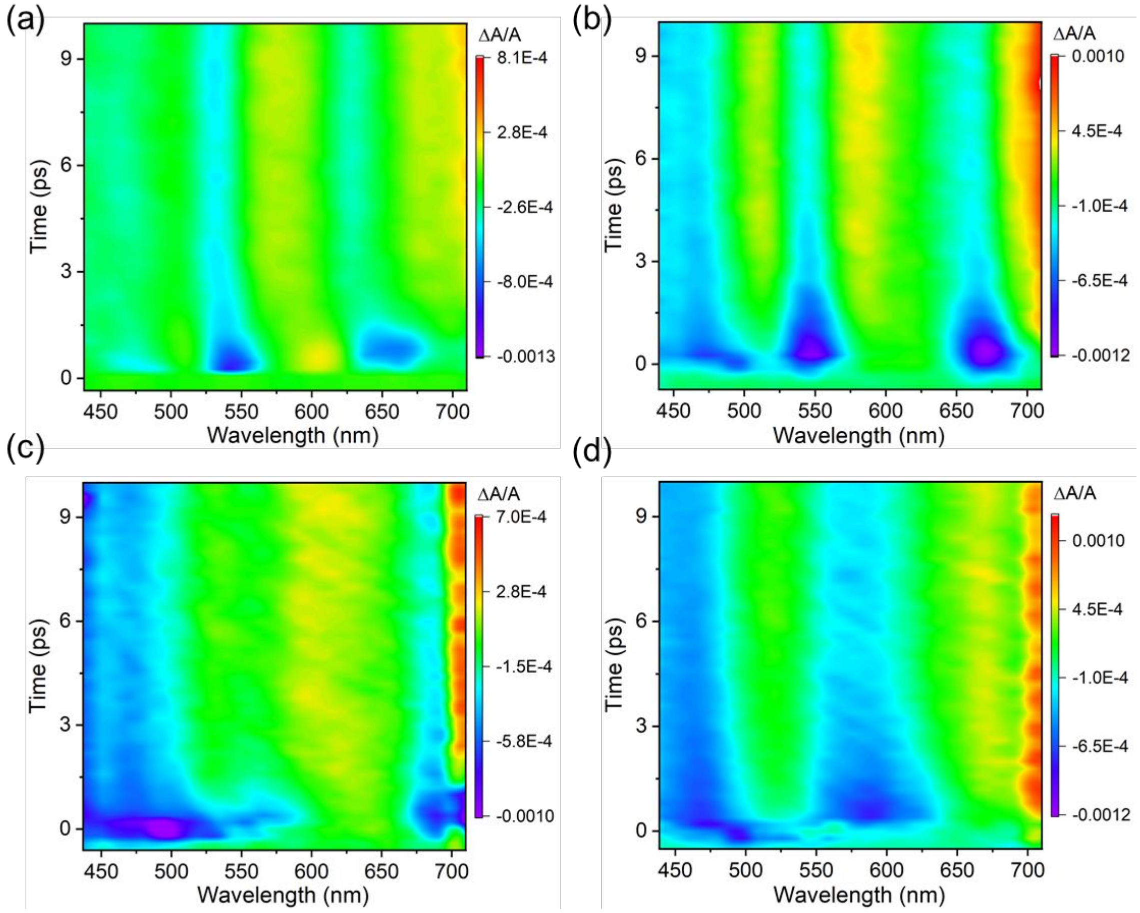

Fig. S17. The short time (9 ps) transient absorption dynamics of (a) WS_2, (b) WSSe (x_{Se}=0.42), (c) WSSe (x_{Se}=0.62) and (d) WSe_2 nanotubes. The diagonals seen at in the yellow color on the transients are an artifact due to the chirp correction, but they do not affect the quantitative analysis.

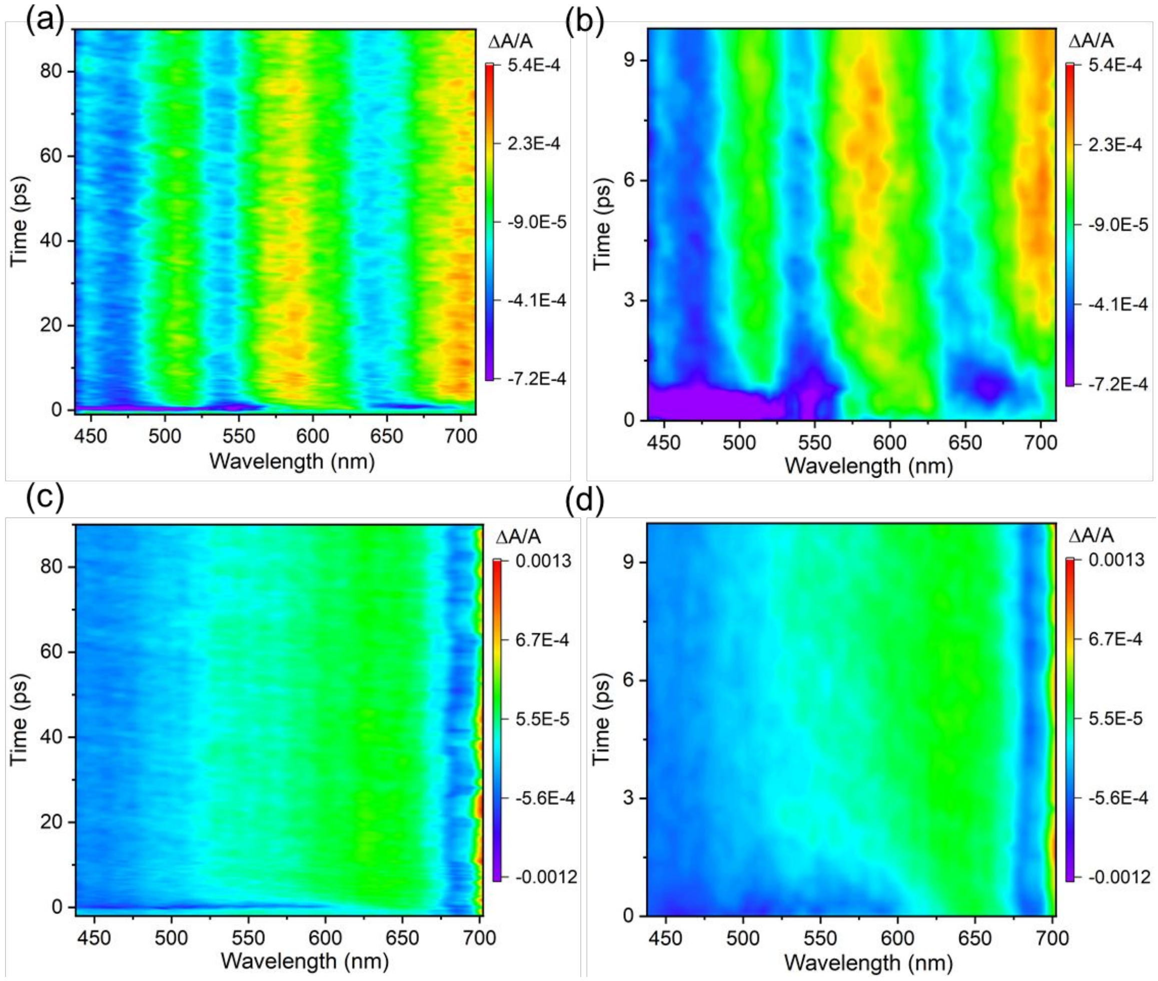

Fig. S18. Transient absorption dynamics (ΔA/A) profile of (a, b) WSSe (x_{Se}=0.18) (c, d) WSSe (x_{Se}=0.77) nanotubes (up to 90 ps and 9 ps delay).

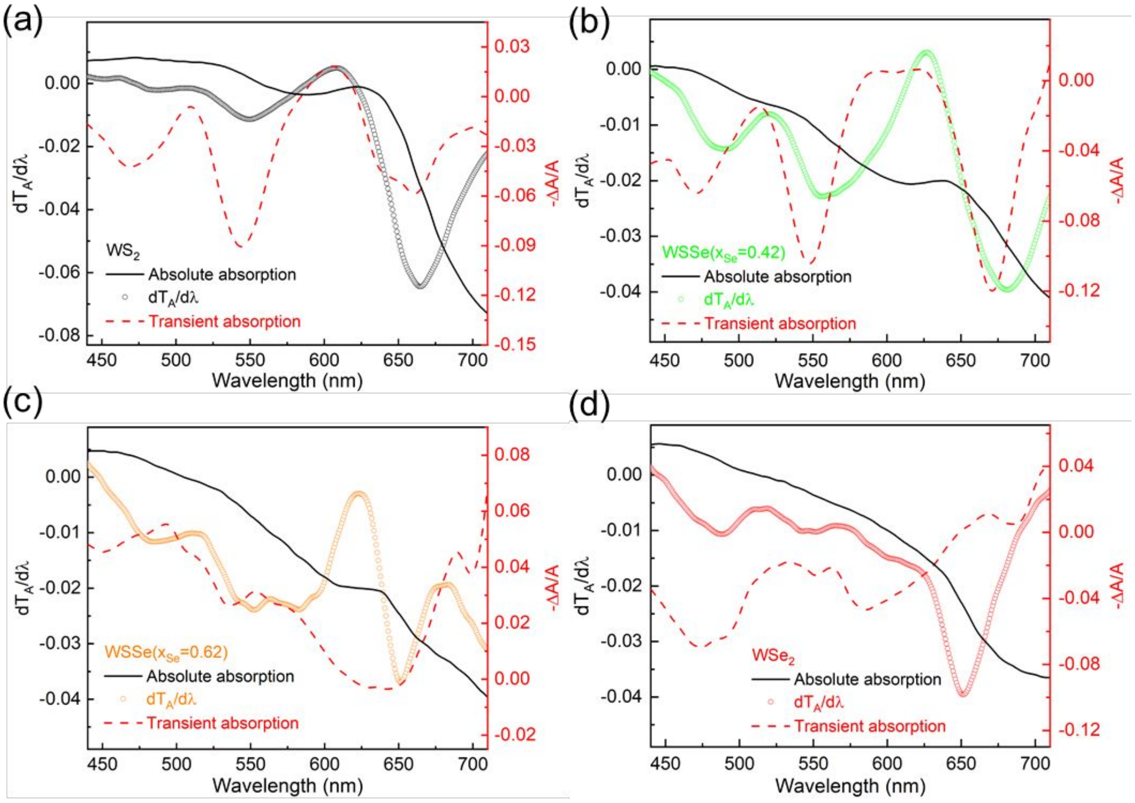

Figure S19. Plots comparing the long delay time (dashed red line) experimental transient absorption (ΔA) and the absolute absorption derivative $\left(\frac{d\varepsilon_A}{d\lambda}\right)$ of steady-state signal (symbol-o) calculated using Equation 1 of (a) WS_2, (b) WSSe (x_{Se}=0.42), (c) WSSe (x_{Se}=0.62) and (d) WSe_2 nanotubes.

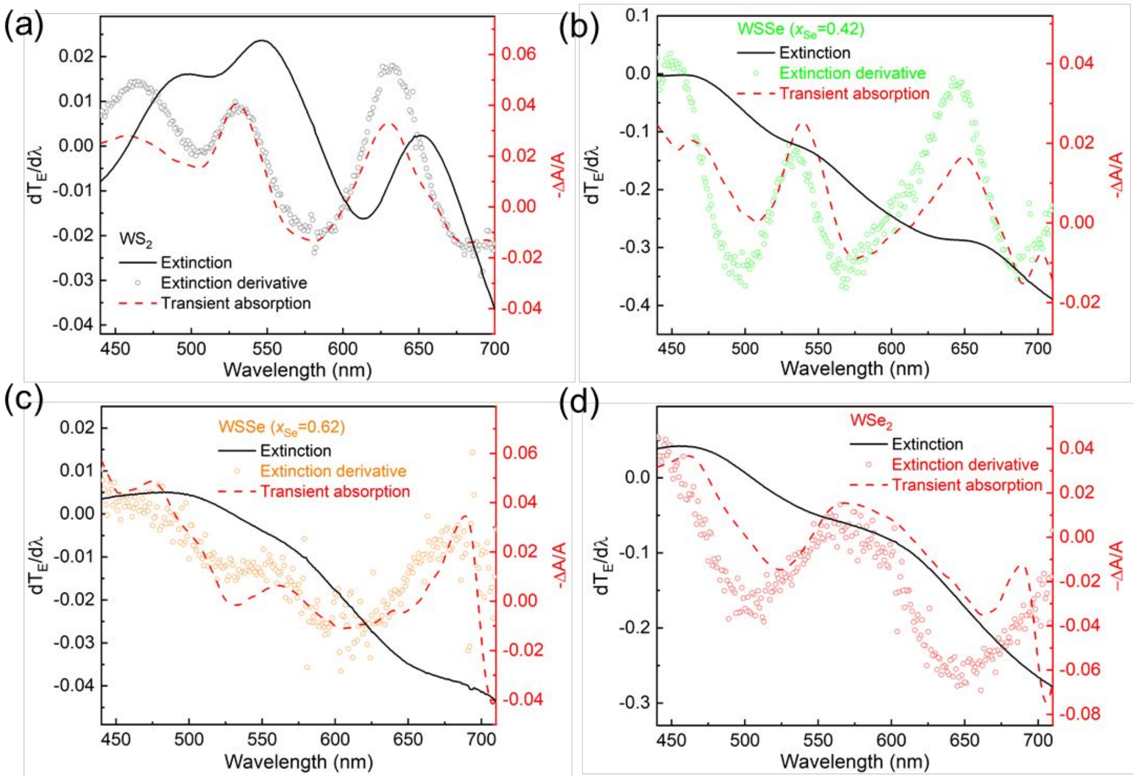

Fig. S20. Plots comparing the long time (dashed red line) experimental transient absorption (ΔA) and the extinction derivative $\left(\frac{d\alpha_E}{d\lambda}\right)$ of steady-state signal (symbol-o) calculated using Equation 1 of (a) WS_2, (b) WSSe (x_{Se}=0.42), (c) WSSe (x_{Se}=0.62) and (d) WSe_2 nanotubes.

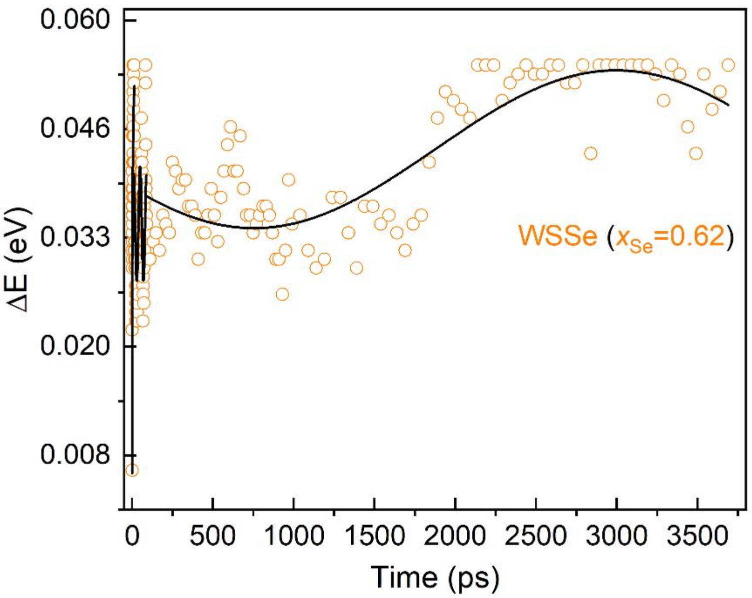

Fig. S21. The dynamics of coherent polaritons in longer time scale (~3ns) of WSSe nanotubes with x_{Se}=0.62.

SI References

(1) Frey, G. L.; Rothschild, A.; Sloan, J.; Rosentsveig, R.; Popovitz-Biro, R.; Tenne, R. Investigations of Nonstoichiometric Tungsten Oxide Nanoparticles. *J. Solid State Chem.* **2001**, *162* (2), 300-314. DOI: https://doi.org/10.1006/jssc.2001.9319.

(2) Egerton, R. F. *Electron energy-loss spectroscopy in the electron microscope*; Springer Science & Business Media, 2011.

(3) Rogers, D. B.; Shannon, R. D.; Sleight, A. W.; Gillson, J. L. Crystal chemistry of metal dioxides with rutile-related structures. *Inorg. Chem.* **1969**, *8* (4), 841-849. DOI: 10.1021/ic50074a029.
